# Environmental DNA from pumped deep-sea water enables monitoring of deep-sea fish diversity

**DOI:** 10.1038/s41598-026-56998-1

**Published:** 2026-06-12

**Authors:** Takao Yoshida, Masaru Kawato, Shinji Tsuchida, Aya Yamazaki, Yoshihiro Fujiwara, Yuriko Nagano, Akinori Yabuki

**Affiliations:** 1 Deep-Sea Biodiversity Research Group, Marine Biodiversity and Environmental Assessment Research Center, Research Institute for Global Change, Japan Agency for Marine-Earth Science and Technology (JAMSTEC), Yokosuka, Kanagawa Japan; 2 Institute for Extra-cutting-edge Science and Technology Avant-garde Research of Life (X-star), Japan Agency for Marine-Earth Science and Technology (JAMSTEC), Yokosuka, Kanagawa Japan; 3 Research Institute for Marine Technology and Engineering, Japan Agency for Marine-Earth Science and Technology (JAMSTEC), Yokosuka, Kanagawa Japan

**Keywords:** eDNA, Biodiversity, Deep-sea water, Fish metabarcoding, Continuous monitoring, Ecology, Ecology, Ocean sciences

## Abstract

Monitoring deep-sea biodiversity is challenging because conventional methods, such as net-sampling and visual surveys, require substantial logistical effort. Environmental DNA (eDNA) offers an alternative for detecting marine organisms; however, its application in deep-sea ecosystems is limited by the sampling accessibility. Because the influence of long‑pipeline transportation on eDNA is unknown, we compared two deep-sea water sampling methods: pumped deep-sea water (pumped method) and water collected near the intake using a Niskin bottle (Niskin method). Samples were collected from pumping stations in Sagami and Suruga bays, Japan, and baited camera observations were conducted to verify eDNA-based detections. In Sagami Bay, both sampling methods detected similar numbers of fish species. In Suruga Bay, the Niskin method exhibited higher species diversity and steeper accumulation curves than the pumped method. The habitat depth ranges of identified species corresponded to the water intake depths in both bays, supporting the effectiveness of the pumped method. Fish community composition showed substantial overlap between methods, and baited cameras recorded 11 and 17 fish taxa in Sagami and Suruga bays, respectively, with 7 and 10 taxa matching eDNA detections. These findings indicate that eDNA metabarcoding of pumped deep-sea water is a feasible method for monitoring deep-sea fish biodiversity.

**Supplementary Information:**

The online version contains supplementary material available at 10.1038/s41598-026-56998-1.

## Introduction

The deep sea is the largest habitat on Earth, with large biodiversity^1–3^. Monitoring of biodiversity is important for conservation and sustainable management of biological resources. However, biodiversity in the deep sea is still not fully elucidated^1,2,4–6^. The combined impacts of climate change, including warming, acidification, deoxygenation, and altered food inputs, together with anthropogenic activities such as deep-sea trawling, mining, and oil extraction, highlight the urgent need for continuous monitoring of deep-sea biodiversity^7,8^.

As marine fish are the most diverse group of vertebrates, they play key roles in marine ecosystems through food webs, including as top predators at higher trophic levels ^9^. They are sensitive to climate change and human activities such as commercial fishing^10–12^. According to recent estimates, mesopelagic fishes inhabiting depths of 200–1000 m comprise roughly 67–94% of the global fish biomass^13^. Many of these species perform diel vertical migration (DVM), feeding in shallower waters at night and moving to deeper waters during the day. These fish are affected by the effects of climate change, such as ocean warming and deoxygenation^14,15^. Conventional fish biodiversity monitoring is typically conducted using capture-based sampling and/or underwater visual observations. However, it is difficult and time-consuming to conduct continuous deep-sea field surveys of fish using vessels equipped with net-sampling devices and visual observation tools, such as remotely operated vehicles and cameras.

**Fig. 1:**
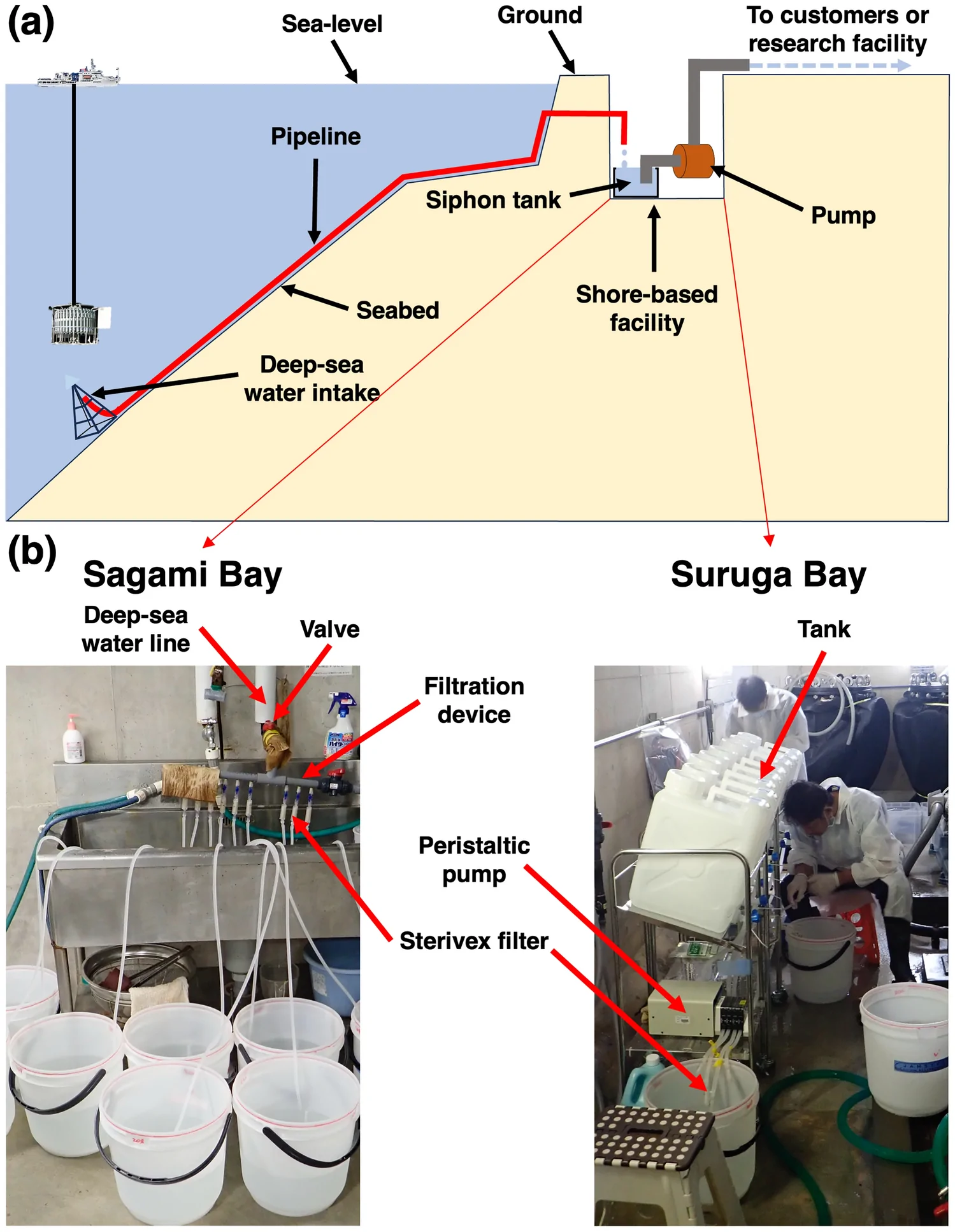
Deep-sea water collection from the pumping station. Schematic drawing of deep-sea water pumping station (**a**) and pumped deep-sea water filtration in Sagami and Suruga Bays (**b**). The pumping station has a deep-sea water intake on the ocean floor, a long pipeline under the sea, and a shore-based facility with a water storage tank. At the shore-based facility, the pipeline’s outlet is below sea level. Deep-sea water is transported using the siphon principle, which is driven by the pressure difference generated by the elevation gradient. Deep-sea water samples were collected at a depth of 10 m above the inlet at the pumping station using Niskin bottles (12 L per bottle) mounted on a CTD frame deployed from a research vessel or fishing boat. The pumped deep-sea water was collected directly from the faucet at the most upstream outlet within the shore-based facilities (**B**). In Sagami Bay, the pumped deep-sea water was directly filtered using a filtration device, whereas in Suruga Bay, it was first collected in a tank and then immediately filtered on site using a peristaltic pump.

Environmental DNA (eDNA) metabarcoding enables the simultaneous detection of multiple species in aquatic environments^16–19^. Owing to its cost-effectiveness and high sensitivity for detecting fish species, eDNA metabarcoding is a powerful tool for biodiversity monitoring compared to conventional methods^20–25^. Recently, eDNA metabarcoding was used to elucidate deep-sea fish biodiversity using deep-sea water pumped from coastal pumping stations^8^. These stations consist of a deep-sea water intake installed on the offshore seafloor, a long subsea pipeline, and a shore-based facility equipped with a water storage tank, and are utilised for commercial and research purposes (Fig. 1). Within the shore-based facility, the pipeline outlet is situated below sea level, and the deep-sea water is pumped up primarily via the siphon principle, driven by the pressure differential resulting from the height change. The term ‘pumped deep-sea water’ in this study refers to water collected directly from the most upstream outlets, such as faucets, at the coastal pumping stations. A previous study using pumped deep-sea water evaluated optimal filtration volumes, thereby identifying conditions that enhance the precision of eDNA metabarcoding analysis for detecting rare species and avoiding PCR inhibition^8^. Hence, pumped deep-sea water can serve as an effective resource for monitoring near-shore deep-sea environments which may be influenced by climate change and anthropogenic activities.

Deep-sea water pumping stations are located in Sagami and Suruga bays (inlet depth of Sagami Bay, 800 m; Suruga Bay, 400 m), Japan. The seafloor topography in both bays is characterised by steep slopes, making them suitable locations for deep-sea biodiversity monitoring. Deep-sea water is principally pumped continuously to the onshore pumping stations, facilitating accessible and immediate on-site sampling at any time without the requirement for research vessels equipped with CTD-mounted Niskin bottles. Although pumping is subject to occasional brief interruptions for maintenance or operational reasons, it generally provides a steady supply of deep-sea water. The water is transported from the intakes to the stations through subsea pipelines, the lengths of which are approximately 5 and 3 km for Sagami Bay and Suruga Bay, respectively. The estimated transit time through these pipelines for water is approximately 30 and 60 min for Sagami Bay and Suruga Bay, respectively. Notably, this continuous suction has the potential to integrate eDNA from the water mass surrounding the inlet, whereas the Niskin bottle provides a more localised ‘snapshot’ of the environment at a specific point in time. However, the effect of pumped deep-sea water transported through long pipelines on eDNA is unknown. Therefore, it is essential to evaluate how these different sampling methods affect the results of deep-sea biodiversity monitoring. In this study, we aimed to investigate the effectiveness of eDNA metabarcoding on fish biodiversity and compare the findings between pumped deep-sea water and water 10 m above the inlet of the deep-sea water pumping station using a Niskin bottle. Moreover, a recent study combined fish eDNA metabarcoding and visual imaging to verify the effectiveness of eDNA metabarcoding^26,27^. Therefore, to validate our findings, we conducted the visual observations of live fish using automated baited cameras to increase the reliability of the results.

## Methods

### Deep-sea water sampling

Deep-sea water samples were collected from two deep-sea water pumping facilities located in Akazawa, Shizuoka (facing Sagami Bay; owned by Akazawa Onsen Village, previous owner, DHC Corporation; pumping depth: 800 m) and Shizuoka Prefecture at Yaizu, Shizuoka (facing Suruga Bay; owned by Shizuoka Prefecture; pumping depth: 400 m) along the Pacific coast of Central Japan (Supplementary Fig. 1 and Supplementary Table S1). The inlet of the pumping station in Sagami Bay is situated approximately 2 m above the seafloor. In contrast, in Suruga Bay, the pipeline from the pumping station was ruptured by an earthquake prior to this study, resulting in the intake lying directly on the seafloor. Deep-sea water samples were collected directly from the deep-sea water pumping stations (hereafter, referred to as the “pump method”) and 10 m above the inlet of the deep-sea water pumping station using Niskin bottles (hereafter, referred to as the “Niskin method”). Within each broad sampling period, the sampling dates for the two methods were 1–11 days apart, with all collections performed during the daytime (approximately 9:00 AM to 3:00 PM; Supplementary Table S1). Thus, the influence of DVM on the eDNA profiles is expected to be minimal. Water collection was conducted using a previously reported pump method^8^. The pumped deep-sea water was collected directly from the faucet located at the most upstream outlet within the pumping stations. Based on previously reported filtration volumes^8^, 20 L of deep-sea water per filter in Sagami Bay was directly filtered using a filtration device that utilised the pressure differential resulting from the height difference between the sea level and outlet (Fig. 1). Similarly, at the Suruga Bay pumping station, 10 L per filter was collected in November 2019, February 2020, August 2020, November 2020, and April 2021, stored in a tank, and then immediately filtered on site using a peristaltic pump (Masterflex L/S peristaltic pump with Masterflex L/S multi-channel cartridge pump head and L/S 17G tubing, Masterflex, Radnor, PA, USA) (Fig. 1). We consider the risk of eDNA degradation during transit to be minimal, as the water is transported through a closed pipeline, maintained at stable deep-sea temperatures, and immediately filtered upon reaching the pumping station. For all samples, a Sterivex filter cartridge (0.45-µm pore size, PVDF membrane, gamma irradiated, sterile, SVHVL10RC, Merck KGaA, Darmstadt, Germany) was utilised for filtration. Before use, all filtration equipment used in this study was sterilised by immersion in 1% or 6% chlorine-based commercial bleach (Kao Co., Tokyo, Japan) and washed thoroughly with pumped deep-sea water or ultrapure water. For the Niskin method, water samples were collected 10 m above the inlet at the stations. This method was conducted using 36 Niskin bottles (12 L per bottle) mounted on a CTD frame on the research vessel (RV) *Kaimei* in November 2020 at both stations during the KM20-09 research cruise. In Suruga Bay, additional sampling was conducted on the fishing boat *Chokane Maru* in November 2019, February 2020, August 2020, and April 2021, with three sampling events conducted within a single day in each period, each using a single 12 L Niskin bottle. At *Chokane Maru*, Niskin bottles were sterilised with 6% chlorine-based commercial bleach before collection and immediately covered with a clean bag after collection to prevent contamination from the ship. Based on these optimised filtration volumes for the pumped method^8^, the Niskin method was designed to minimise the volume difference between the two sampling approaches within the physical constraints of the 12 L Niskin bottles. A total of 24 L (two bottles) and 12 L (one bottle) of deep-sea water collected from Sagami and Suruga bays, respectively, were also filtered through Sterivex filter cartridges on board *Kaimei* or at the home port (Yaizu port) of *Chokane Maru* using a peristaltic pump. After filtration by pumped and Niskin methods, approximately 2.0 mL of RNAlater (Thermo Fisher Scientific, Waltham, MA, USA) was immediately added to each cartridge through the inlet to prevent eDNA degradation, and the inlet and outlet were capped. After filtration, the Sterivex cartridges were stored at 4 °C at the stations and *Chokane Maru* or − 30 °C onboard until the samples were returned to the laboratory. All the samples were stored at − 30 °C in the laboratory until eDNA extraction. Negative control samples were used to ensure the validity of our eDNA analysis. As a negative control, during filtration, 20 or 10 L of ultra-purified water (Milli-Q), which was filled in a single-use sterilised plastic bag (Sekisui Seikei Co., Ltd., Osaka, Japan), was passed through a Sterivex filter cartridge with a peristaltic pump. An infiltrated Sterivex filled with RNAlater was used as a negative control (eDNA elution blank; EB) at each collection site.

### DNA extraction, library preparation, and sequencing

eDNA extraction from the Sterivex cartridge, library preparation including two-step PCR for metabarcoding of fish in eDNA using two universal primer sets, and MiSeq sequencing were performed according to previous studies^8,17,28^. For eDNA extraction, the filtration membrane within Sterivex filter cartridges was removed, cut into pieces for lysis, and eDNA was extracted as described previously^28^. Library preparation, including a two-step PCR for fish metabarcoding, followed a previous method^28^ based on Miya et al. 2015^17^. In the first round, a multiplex PCR assay targeted the mitochondrial 12S rRNA gene using two universal primer sets. The primer sequences with linkers were as follows: 5ʹ-ACACTCTTTCCCTACACGACGCTCTTCCGATCT-NNNNNN-GTCGGTAAAACTCGTGCCAGC-3ʹ (MiFish-U-Forward), 5ʹ-ACACTCTTTCCCTACACGACGCTCTTCCGATCT-NNNNNN-GTTGGTAAATCTCGTGCCAGC-3ʹ (MiFish-E-Forward), 5ʹ-GTGACTGGAGTTCAGACGTGTGCTCTTCCGATCT-NNNNNN-CATAGTGGGGTATCTAATCCC-3ʹ (MiFish-U-Reverse), 5ʹ-GTGACTGGAGTTCAGACGTGTGCTCTTCCGATCT-NNNNNN-CATAGTGGGGTATCTAATCCTAGTTTG-3ʹ (MiFish-E-Reverse)^17^. Comprehensive technical details regarding eDNA extraction, library preparation, and sequencing are provided in the Supplementary Method. The raw sequencing data were deposited in the NCBI sequence read archive (SRA) under the BioProject number PRJDB18791. Amplicon raw sequence data processing was conducted using PMifish ver. 2.4.1 (available at https://github.com/rogotoh/PMiFish.git)^29,30^ and USEARCH version 11.0.667^31^. Initially, raw paired-end reads were merged, excluding those with low quality (Q score < 2) or short length (< 100 bp) after tail trimming. Merged reads with discrepancies exceeding 5 bp in the aligned region were discarded. Following primer sequence removal, reads with an expected error rate above 1% or those shorter than 120 bp were filtered out. The sequences were then dereplicated to obtain unique sequences, with singletons, doubletons, and tripletons eliminated to prevent false positives^31,32^. These unique sequences were processed to generate amplicon sequence variants (ASVs), with chimeric and erroneous sequences removed^33^. Furthermore, sequences for each sample were filtered to remove all ASVs that occurred only once in the study, as well as samples containing only a single ASV, to avoid outliers that might bias the results^34^.

Taxonomic assignment of these ASVs followed a previously established protocol to define molecular operational taxonomic units (MOTU)^29,30^. ASV sequences with identities greater than 98.5% that formed a monophyletic clade with a reference sequence were assigned that species name. ASV sequences with identities between 80% and 98.5% were assigned the prefix “U98.5_” followed by the name of the species with the highest identity. ASVs with sequence identities lower than 80% were removed from the taxonomic assignment. Each ASV in a monophyletic clade of species belonging to a particular genus was assigned the genus name followed by “sp.” with a sequential number (e.g., *Lepidotrigla* sp. 1, sp. 2). Each ASV in a monophyletic clade of a particular family but not in a particular genus was assigned the family name followed by “sp.” with a sequential number (e.g., Synaphobranchidae sp. 1, sp. 2 or U98.5_Myctophidae sp. 1, U98.5_Myctophidae sp. 2) (Supplementary Tables S2-1, S2-2, and S2-3). Hereafter, the terms “MOTU" and “species” are used interchangeably. All ASV sequences generated in this study are provided in the Supplementary Data.

### Statistical analysis

All statistical analyses were conducted using R 4.4.2^35^, run in RStudio v2024.12.0 + 467^36^. To assess the effect of sampling effort on the number of species detected, accumulation curves and Chao indices were calculated and plotted for the number of ASVs detected by the two water collection methods at each site using the iNEXT v3.0.1 package in R^37^. The difference between the accumulation curves for the two water collection methods was compared using the "EcoTest.sample" function of rareNMtests v1.2 package in R^38^. Venn diagrams showing the differences in species composition between the water collection methods at each site were illustrated using the eulerr v7.0.2 package in R^39,40^. Chord diagrams comparing fish MOTUs between the different collection methods were constructed using the circlize v0.4.16 package in R^41^. Hierarchical clustering based on the Jaccard index, which uses presence-absence data, was constructed using the unweighted pair group method with arithmetic mean (UPGMA) via the hclust function in R. The differences in fish composition between the water collection methods were visualised using three-dimensional non-metric multidimensional scaling (NMDS) analysis based on the presence/absence of MOTUs in individual samples from Sagami and Suruga bays. NMDS analysis was performed using the Jaccard distance with the metaMDS function in the vegan v2.6.8^42^ package in R, with three dimensions (*k* = 3). Permutational multivariate analysis of variance (PERMANOVA) tests were performed using the adonis function in vegan to assess fish community structures between the two water collection methods. For PERMANOVA, Jaccard similarity matrix was used and statistical values with 99,999 permutations were calculated. Significant differences in dispersion between the two water collection methods were tested using PERMDISP to aid in the interpretation of PERMANOVA results, implemented via the betadisper function in the vegan package. To account for temporal variation across the five distinct sampling periods in Suruga Bay, a multifactorial PERMANOVA was also performed with sampling method and sampling period as factors. The analysis was based on a Jaccard distance matrix with 99,999 permutations, and marginal effects were calculated to assess the independent contribution of each factor. We compared the differences in the number of ASVs detected by the two water collection methods at each site using the Mann–Whitney U test in the coin package v1.4–3 in RStudio. Pearson’s correlation coefficient analysis was performed to evaluate the effect of filtration volume on the number of detected ASVs in RStudio. To account for the potential random effect of sampling period in Suruga Bay, a generalised linear mixed model (GLMM) was applied to the ASV richness data using the “glmer” function in the lme4 package (version 2.0–1)^43^. In this model, the number of detected ASVs was used as the response variable, the sampling method as a fixed effect, and the sampling period as a random effect, assuming a Poisson distribution. To evaluate whether the two sampling methods consistently captured the biodiversity of the fish community at the sampling depth and to investigate potential biases in species detection, we analysed the habitat depth ranges of MOTUs identified at the species level using the habitat depth records available in FishBase. A Kruskal–Wallis test was performed to determine the differences in habitat depth of fish species detected by the two water collection methods using the coin package v1.4–3 in R. Differences were considered statistically significant at *p* < 0.05. Graphics were constructed using ggplot2 v3.5.1 in R^44^.

### Baited camera system

Fish observations were conducted using a baited camera system to compare results with fish biodiversity detected by eDNA analysis. During the KM20-09 cruise of R/V *Kaimei* in November 2020, free-fall baited camera systems, called BCMs, were deployed around the inlet of the deep-sea water pumping stations in Sagami (800 m depth) and Suruga (400 m depth) bays and recovered by *KM-ROV* 1–2 days later. One cast of two BCMs in Sagami Bay and two casts of two BCMs in Suruga Bay were conducted (Supplementary Table S3), and an approximately 6–12 h video was acquired at the bottom of each cast.

In November 2019, February 2020, August 2020, and April 2021, three BCMs moored with a rope and a buoy were deployed at the inlet of the deep-sea water pumping station in Suruga Bay and recovered via the rope using the fishing boat *Chokane Maru* (Supplementary Table S3). A total of 26 casts were conducted to obtain 2–8 h videos for each cast.

The BCM was equipped with a digital still camera (DSC-RX0; Sony, Tokyo, Japan), and two LED flashlights (model no. OL0183; KC Fire, Guangdong, China), three aluminium alloy housings for the camera and flashlights, an electromagnetic current profiler (Infinity-EM; JFE Advantech, Nishinomiya, Japan); a miniature salinity, temperature, and depth logger (DST-CTD; Star-Oddi, Garðabær, Iceland), an acoustic release transponder (STE; Kaiyo Denshi, Tsurugashima, Japan), an ROV Homer (Sonardyne International, Yateley, UK), a stainless-steel bait cage containing approximately 1.0 kg of frozen mackerel, a syntactic foam (TG2000; Trelleborg AB, Trelleborg, Sweden), and a stainless steel frame. All the data, including video footage, were recovered upon retrieval of the system. All animals observed by the video camera were identified to the most specific taxonomic level possible, ideally to the species level, but at least to the family level. The population density (N) of each species appearing in the videos was estimated using the first arrival time (Tarr) according to the method outlined by Priede and Merrett (1996)^45^. These estimations were utilised for comparative validation with the eDNA results. Although mackerel was utilised as bait for the camera observations, detections of Scombridae (taxa closely related to mackerel) were retained in the final eDNA dataset based on the following validation criteria. Scombridae eDNA was confirmed in samples collected prior to the deployment of the baited camera systems, specifically in Sagami Bay in November 2020 and in Suruga Bay in August 2020 and August 2021. This indicates that their presence was independent of the experimental bait. Therefore, these eDNA detections were considered representative of the actual environmental profile and were included in all subsequent analyses to ensure data integrity.

## Results

### Sample collection and sequence analysis

In total, 33 eDNA samples were collected from Sagami Bay, and 12 and 21 eDNA samples were obtained using the Niskin and pump methods, respectively (Supplementary Table S1); 67 eDNA samples were collected from Suruga Bay, of which 36 and 31 eDNA samples were obtained using the Niskin and pump methods, respectively (Supplementary Table S1). Fish 12S rRNA gene fragments were successfully amplified and sequenced from all the samples (detailed data are presented in Supplementary Table S1). The number of raw reads for each library ranged from 105,221 to 244,259, with an average of 150,677 reads (Supplementary Table S1). After sequence processing, the number of reads identified as fish ranged from 52,940 to 157,731 per sample, with an average of 95,095 (Supplementary Table S1). The negative controls showed no detectable DNA concentrations and no PCR amplification.

### Comparison of fish species detected by pump and niskin methods

**Fig. 2:**
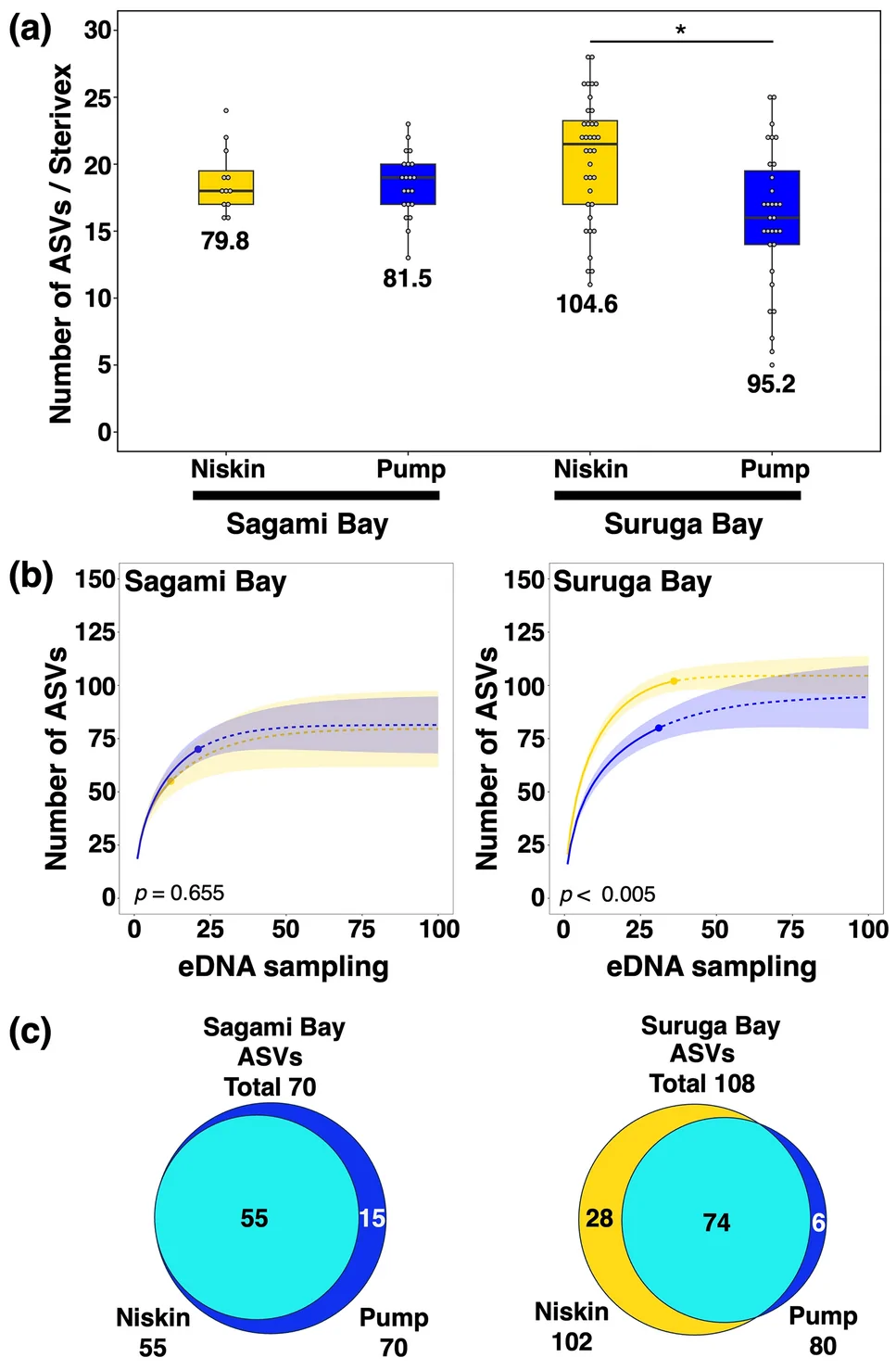
Comparison of the number of fish ASVs detected using the Niskin and pump methods. Boxplots with circles indicate the number of ASVs detected per eDNA sample (**a**). Boxes and thick horizontal bars represent the interquartile range and median values, respectively. The asterisk indicates a significant difference in the number of ASVs between the two water collection methods based on the Mann–Whitney U test (*p* < 0.05). The values in each plot indicate the alpha diversity (Chao index). Accumulation curves of the number of ASVs s detected by the two water collection methods (**b**). Solid and dotted lines indicate sample-size-based rarefaction and extrapolated sampling plots, respectively. Deep-sea water was collected by Niskin method (yellow) and pump method (blue). The shaded region shows the 95% confidence intervals. EcoTest was used to test the significance of the differences between the rarefaction curves of the methods, and the results (*p*-values) are shown in each panel. Venn diagrams show the number of fish ASVs detected by the Niskin method specific (yellow), the pump method specific (blue), and those shared by both methods (light blue) in Sagami and Suruga bays (**c**).

In Sagami Bay, the number of ASVs detected per eDNA sample ranged from 16 to 24 (mean = 18.75) using the Niskin method and from 13 to 23 (mean = 18.48) using the pump method, with no significant difference between the 2 collection methods (Mann–Whitney U test; *p* = 0.9781; Fig. 2a). Furthermore, to evaluate the effect of the differences in filtration volumes between the Niskin and pump methods on the detection ASV number, we performed Pearson’s correlation coefficient analysis. No significant correlation was found in Sagami Bay (*p* > 0.05). Conversely, Suruga Bay showed a significant difference between the 2 collection methods (Mann–Whitney U test;* p* < 0.05), with ASVs ranging from 11 to 28 (mean = 20.44) using the Niskin method and from 5 to 25 (mean = 15.97) using the pump method (Fig. 2a). In Suruga Bay, a significant positive correlation was observed between filtration volume and the number of detected ASVs (Pearson’s correlation coefficient, *p* < 0.05). To account for the potential influence of temporal variability, we conducted a generalised linear mixed model (GLMM) with the sampling date treated as a random effect. This analysis revealed that the pump method had a negative coefficient compared to the Niskin method, and the difference in detected species richness between the two sampling methods was significant (*p* < 0.05). Alpha diversity (species richness across water collection methods) ranged from 79.8 to 104.6 (Fig. 2a). In Sagami Bay, no significant differences were observed between the accumulation curves of the detected ASVs for the two collection methods (EcoTest, *p* = 0.875; Fig. 2b). However, in Suruga Bay, a significant difference was observed, with the number of ASVs detected using the Niskin method being higher than those detected using the pump method (EcoTest, *p* < 0.005; Fig. 2b).

**Fig. 3:**
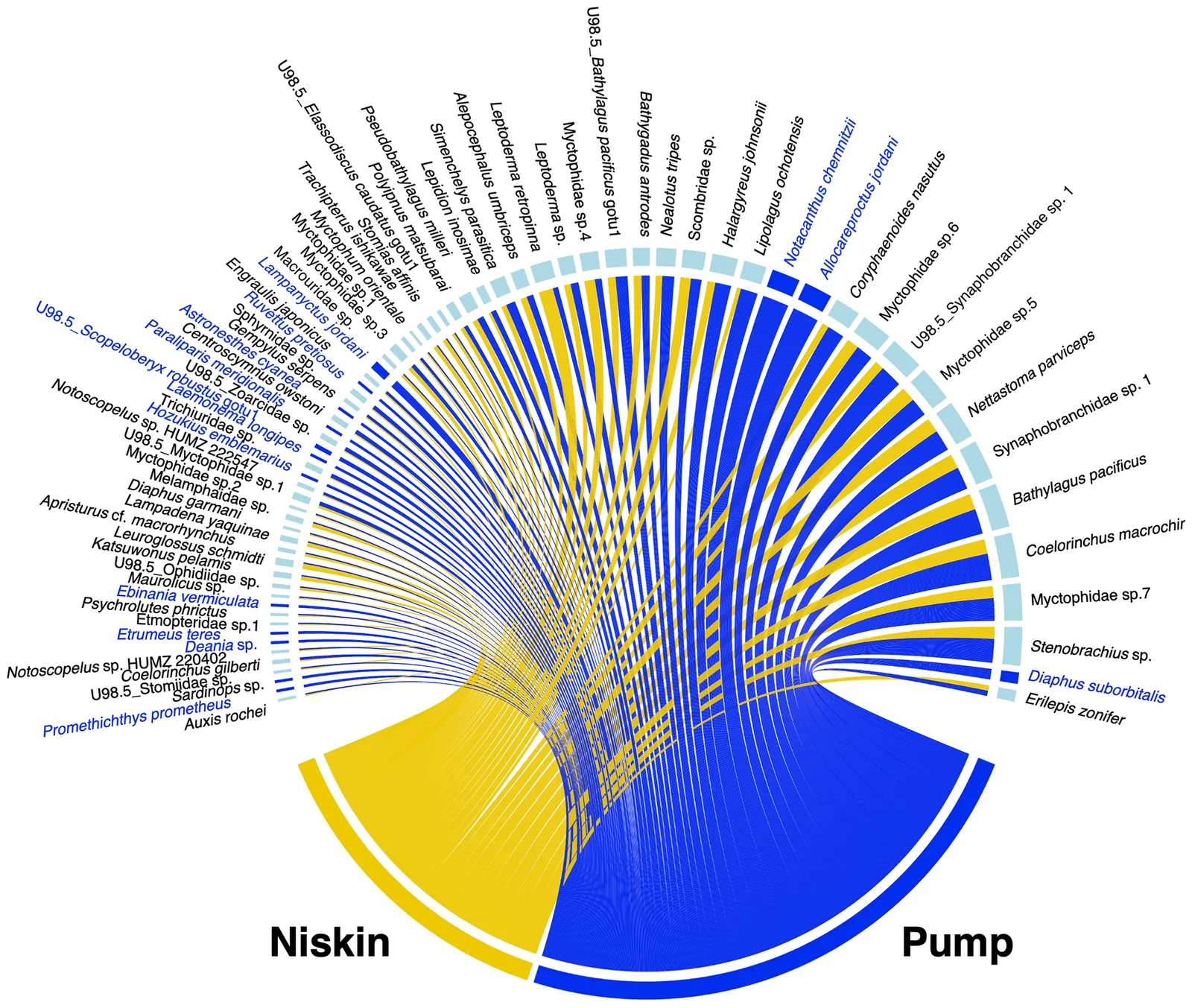
Composition of fish MOTUs detected by the Niskin and pump method in Sagami Bay. The detected MOTUs in Sagami Bay. The chord diagram based on sequence presence indicates the mapped detected fish MOTUs by the Niskin method (yellow) and pump method (blue). The outer columns represent the MOTUs with pump method specific (blue), and shared by both methods (light blue). Pump method specific detected species was indicated as blue. The MOTU order was determined by clustering using the unweighted pair group method with the arithmetic mean.

**Fig. 4:**
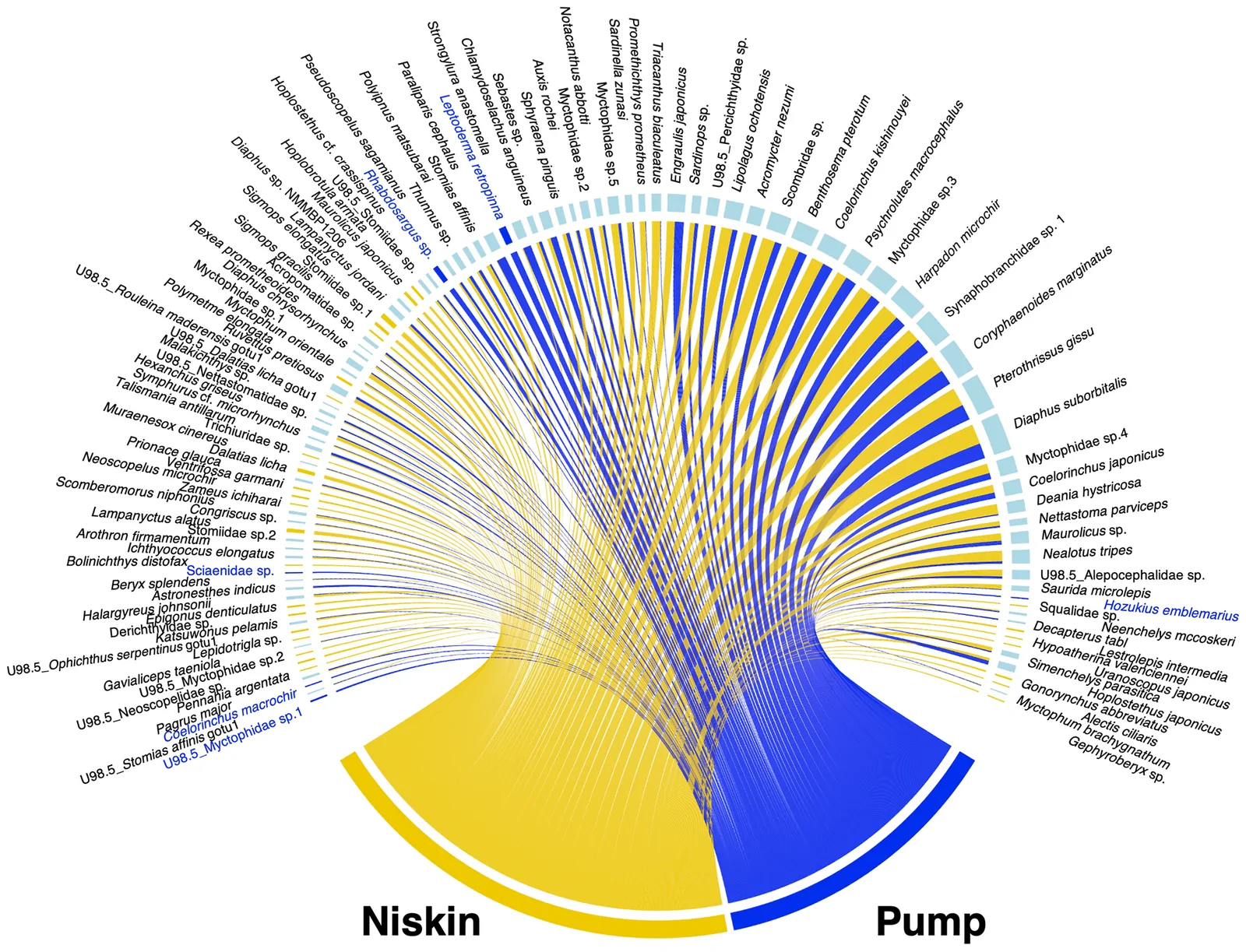
Composition of fish MOTUs detected by the Niskin and pump method in Suruga Bay. The detected MOTUs in Suruga Bay. The chord diagram based on sequence presence indicates the mapped detected fish MOTUs by the Niskin method (yellow) and pump method (blue). The outer columns represent the MOTUs with Niskin method specific (yellow), pump method specific (blue), and shared by both methods (light blue). Pump method specific detected species was indicated as blue. The MOTU order was determined by clustering using the unweighted pair group method with the arithmetic mean.

A total of 148 ASVs representing fish were identified across both bays (Supplementary Table S2-1). Among them, 96 were identified at the species level, 15 at the genus level, 20 at the family level, and 17 as sequences with identities ranging from 80% to 98.5% (Supplementary Table S2-1). In Sagami Bay, 70 ASVs were detected. Among them, 15 ASVs were identified only by the pump method, with occurrence rates ranging from 6.1% to 60.6% (2 to 20 filter samples), whereas 55 ASVs were detected by both sampling methods, with occurrence rates ranging from 6.1% to 93.9% (2 to 31 filter samples; Fig. 2c, Supplementary Fig. 2a, and Supplementary Table S2-2). In Suruga Bay, 108 ASVs were detected, with 28 ASVs identified by the Niskin method at rates of 2.99% to 11.94% (2 to 8 filter samples); 6 ASVs were identified using the pump method at rates of 2.99% to 17.91% (2 to 12 filter samples); and 74 ASVs were detected by both sampling methods at rates of 2.99% to 94.03% (2 to 63 filter samples; Fig. 2c, Supplementary Fig. 2b, and Supplementary Table S2-3). These results indicate that the proportion of ASVs shared between the two collection methods was 78.6% in Sagami Bay and 68.5% in Suruga Bay. The interrelationships between the two collection methods and the composition of the MOTUs are represented in a chord diagram (Fig. 3 and 4). Among the detected MOTUs, most with > 50% occurrence ratios in both bays were identified by both sampling methods, except for MOTUs corresponding to *Allocareproctus jordani* and *Notacanthus chemnitzii* in Sagami Bay, which are bathydemersal and benthopelagic fish, respectively, and were only detected by the pump method (Fig. 3 and 4, and Supplementary Figs. 2, 3, and 4).

**Fig. 5:**
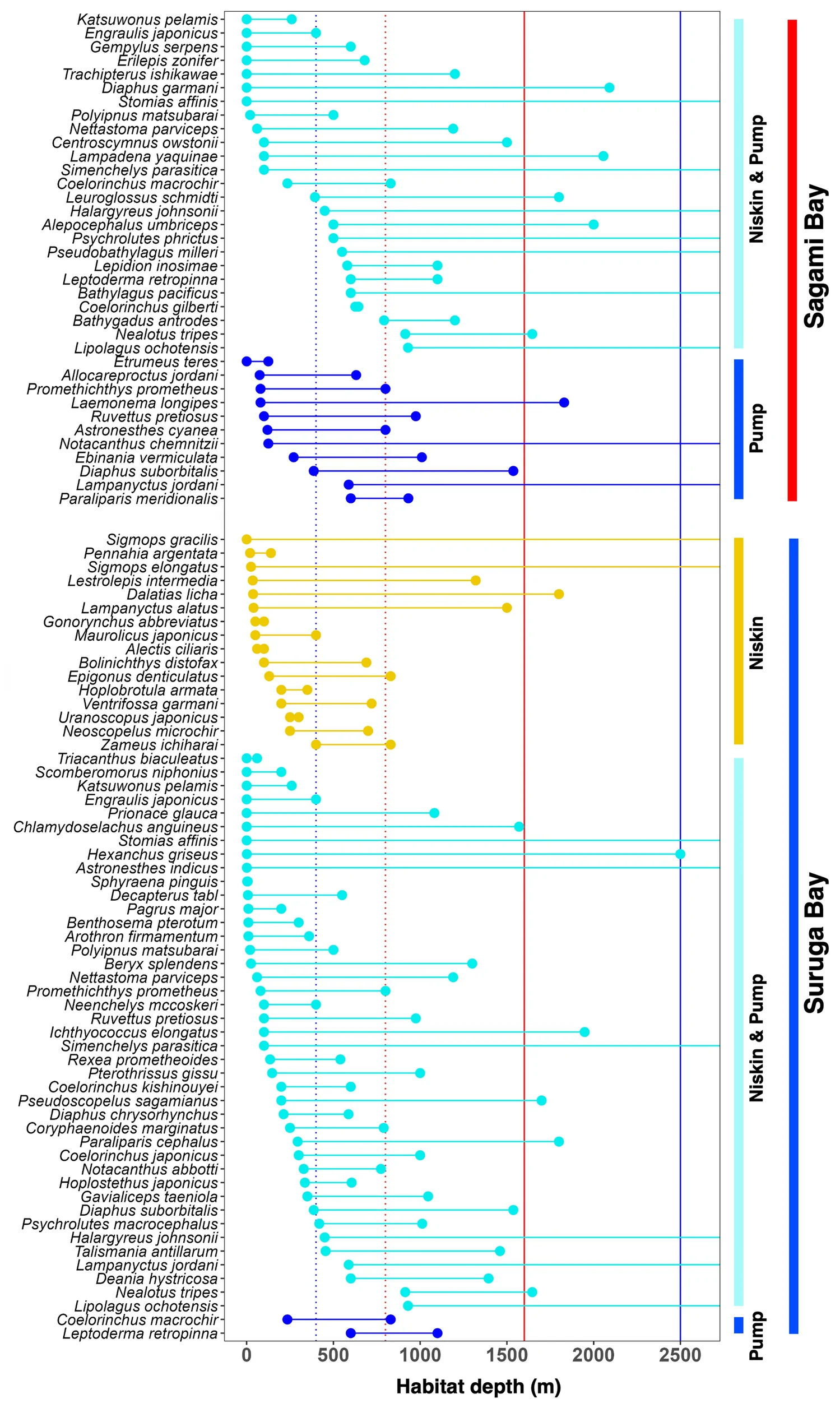
Range of habitat depths for fish species-level taxa. The shallowest and deepest habitat depths for MOTU fish species with available records in FishBase (https://www.fishbase.se/home.html) were plotted. Species specific to the Niskin method (yellow), specific to the pump method (blue), and shared by both methods (light blue) in Sagami and Suruga bays. The inlet depths of the deep-sea pumping stations in Sagami and Suruga bays are shown as dotted red and blue lines, respectively. The maximum depths of Sagami and Suruga bays are indicated by solid red and blue lines, respectively. There was no evidence in either bay that habitat depths (shallow, intermediate, deepest) of fish species differed significantly between the two collection methods (*p* < 0.05, Kruskal–Wallis test).

Most MOTUs exhibited habitat depth ranges that were consistent with the intake depths of the respective pump stations (Fig. 5). However, the habitat depths of several MOTUs, including *Nealotus tripes* and *Lipolagus ochotensis* in Sagami Bay, and *Psychrolutes macrocephalus*, *Halargyreus johnsonii*, *Talismania antillarum*, *Lampanyctus jordani*, *Deania hystricosa*, *Nealotus tripes*, *Lipolagus ochotensis*, and *Leptoderma retropinna* in Suruga Bay were below the inlet depth (Fig. 5). A Kruskal–Wallis test indicated that the distribution of habitat depths (representing the shallowest, intermediate, and deepest recorded depths of the detected species) did not differ significantly between the two collection methods in either bay (*p* > 0.05). These results suggest that both methods consistently detected fish species with similar depth-habitat characteristics.

### Fish composition

**Fig. 6:**
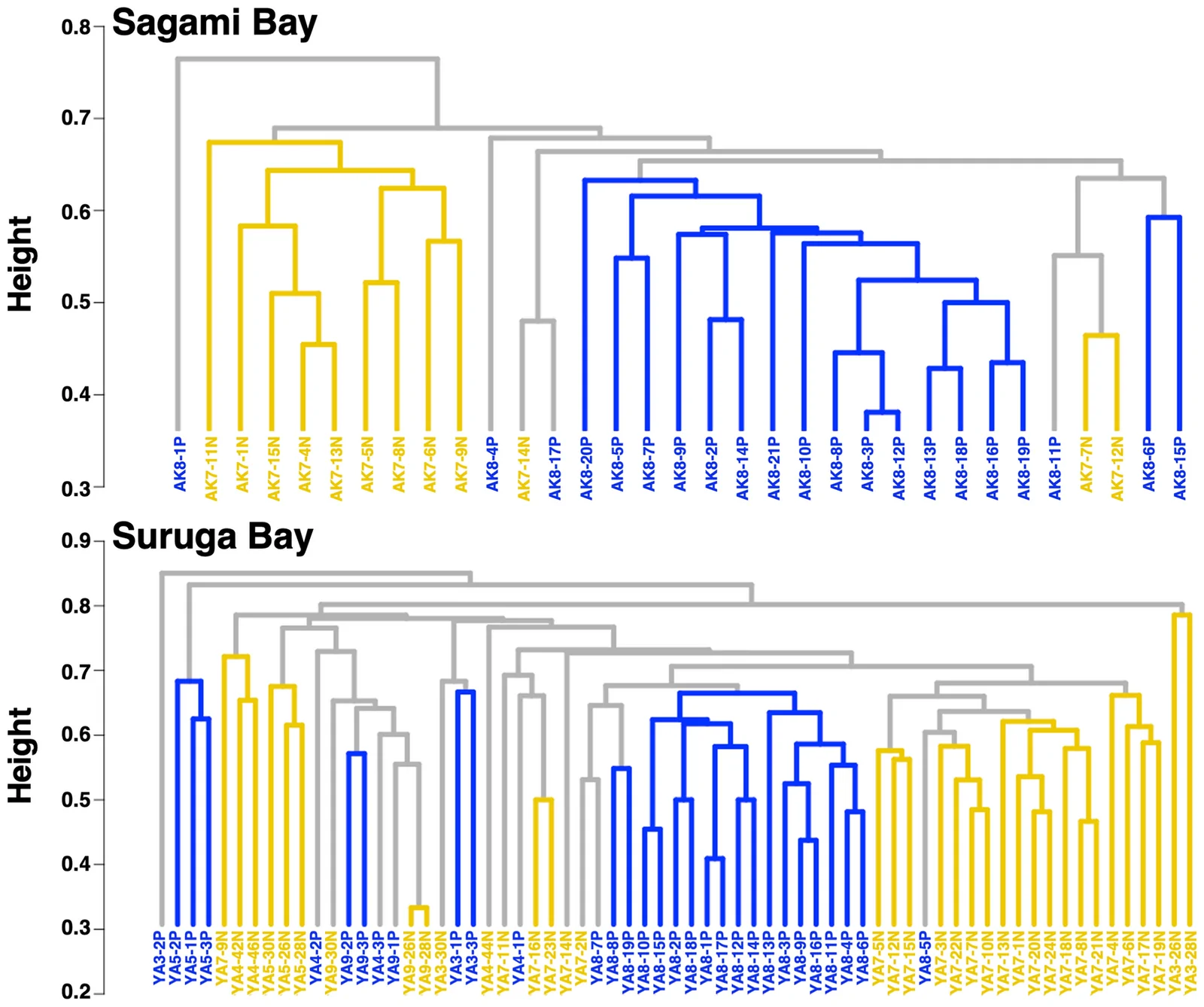
Hierarchical clustering dendrograms of fish between the two water collection methods at Sagami and Suruga bays. Clustering based on the presence/absence of MOTU reads was performed using the unweighted pair group method with arithmetic mean (UPGMA) and Jaccard index. Yellow and blue characters indicate eDNA samples collected by the Niskin and pump method, respectively. Coloured branches indicate the clusters formed within the same collection methods: Niskin method (yellow) or pump method (blue), whereas gray branches indicate clusters that were not formed within the same collection method.

**Fig. 7:**
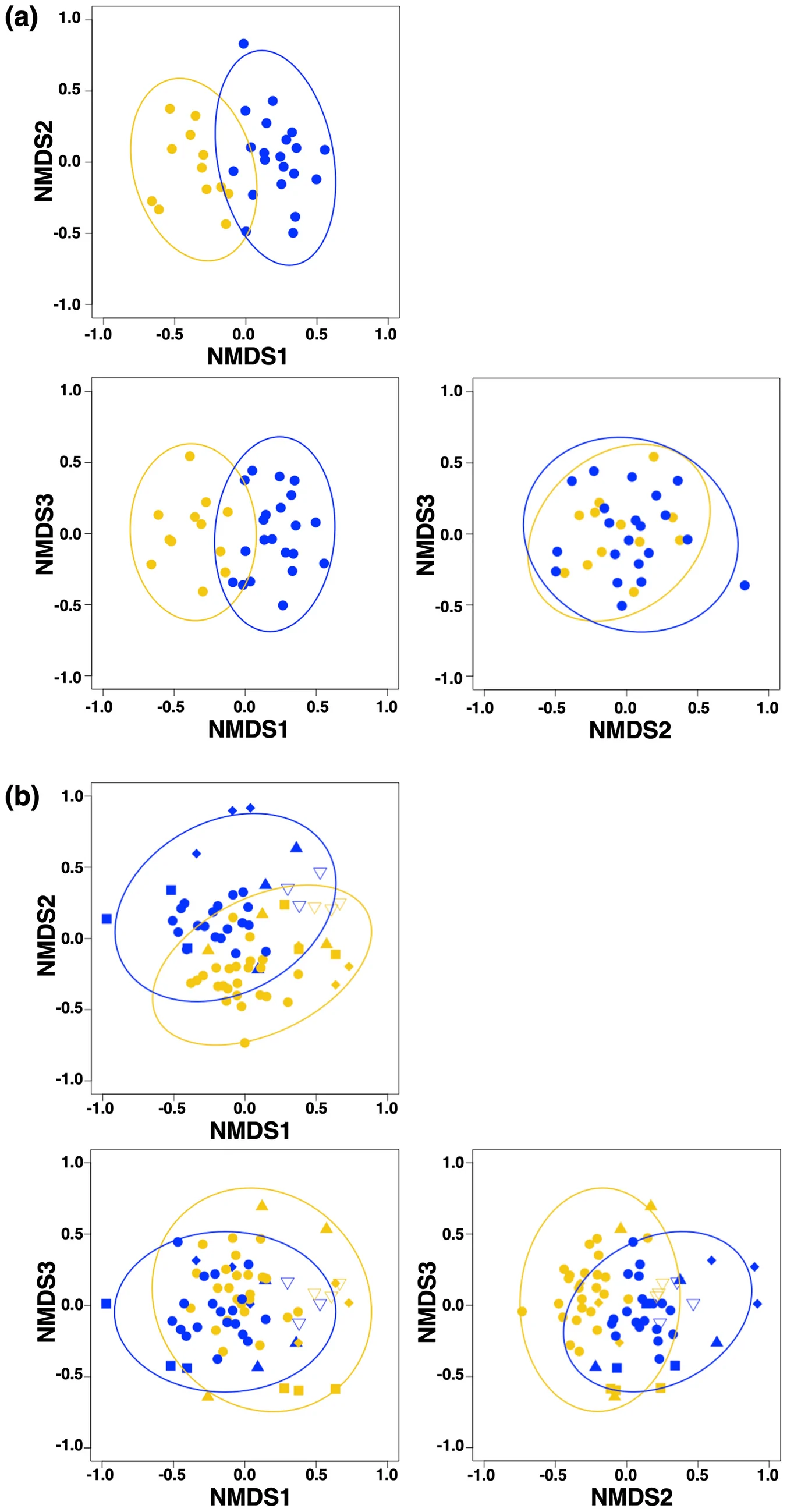
Non-metric multidimensional scaling (NMDS) plots of fish community between the water collection methods in Sagami and Suruga bays. The analyses were performed using the presence/absence data of MOTU reads and Jaccard distance. In Sagami Bay, yellow and blue circles indicate eDNA samples collected in November 2020 using the Niskin and pump methods, respectively (**a**). In Suruga Bay, symbols represent sampling periods: squares (November 2019), triangles (February 2020), diamonds (August 2020), circles (November 2020), and open inverted triangles (April 2021), with yellow and blue indicating samples collected using the Niskin and pump methods, respectively (**b**). Ellipses represent the 95% confidence level based on the centroid for each water collection method. The NMDS stress values for Sagami and Suruga bays were 0.1779 and 0.2033, respectively.

To assess the similarity in fish composition in eDNA samples between the two collection methods, hierarchical cluster analysis using UPGMA was conducted based on the Jaccard index using presence-absence data. The resulting cluster plots for both bays revealed that several branches from the same collection method clustered together (Fig. 6). Several other branches were clustered using both the collection methods (Fig. 6). Overall, similar detection patterns of fish species were observed among the clustered branches (Supplementary Figs. 3 and 4). In the NMDS analysis, clusters of fish taxonomic compositions corresponding to the same collection method were observed in both bays. These clusters overlapped within the 95% confidence interval ellipsoids for the two collection methods (Fig. 7). The NMDS stress values in Sagami and Suruga bays were 0.1779 and 0.2033, respectively. PERMANOVA based on Jaccard distance indicated significant differences in fish community composition between the two collection methods in both Sagami (pseudo*-F*_1,31_ = 4.2743, *R*^*2*^ = 0.12117, *p* < 0.001) and Suruga (pseudo*-F*_1,65_ = 4.1832, *R*^*2*^ = 0.06047, *p* < 0.001) bays. Conversely, PERMDISP revealed no significant difference in multivariate dispersion between the two methods in either bay (Sagami Bay: pseudo*-F*_1,31_ = 0.04, *p* = 0.84; Suruga Bay: pseudo*-F*_1,65_ = 0.3352, *p* = 0.5646). In Suruga Bay, eDNA sampling was conducted across five distinct periods. To account for these varying sampling periods, we performed a multifactorial PERMANOVA including both sampling method and sampling period as factors. The results showed that although the sampling method had a significant effect (pseudo*-F*_1,61_ = 4.80, *R*^*2*^ = 0.061, *p* < 0.0001), the sampling period showed a larger proportion of variation in community composition than the sampling method (pseudo*-F*_4,61_ = 3.38, *R*^*2*^ = 0.170, *p* < 0.0001). However, the effect of the sampling period was reduced when the data were analysed by pooling samples collected using the same method within each sampling period. In Suruga Bay, community composition was not significantly affected by the collection method when eDNA data collected using the same method within each sampling period were pooled prior to analysis (PERMDISP: pseudo*-F*_1,8_ = 0.349, *p* = 0.571; PERMANOVA: pseudo*-F*_1,8_ = 1.0627, *R*^*2*^ = 0.11726, *p* = 0.333).

### Baited camera observation and eDNA detection

**Fig. 8:**
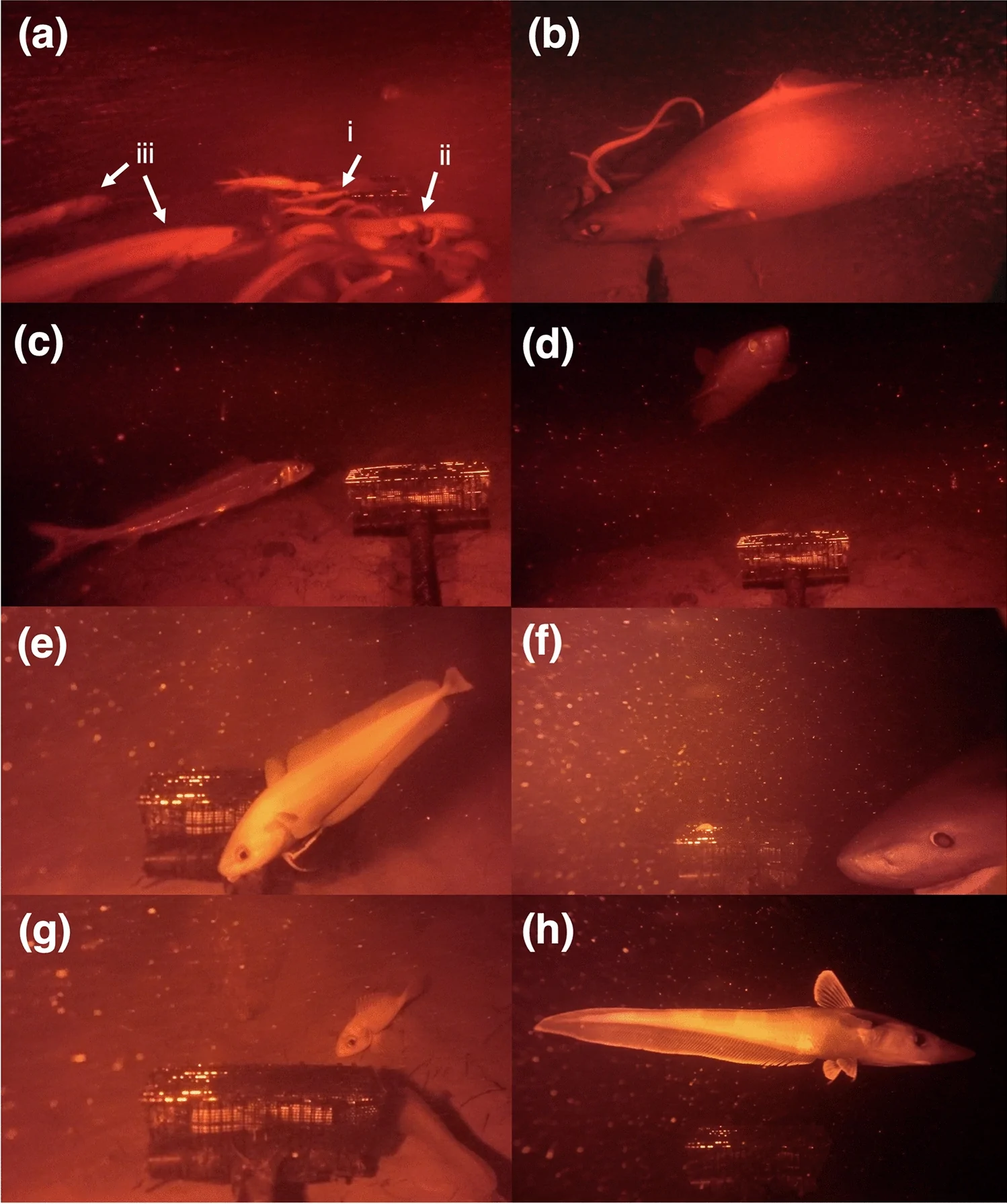
Observed fishes captured by baited cameras in Sagami (**a**, **b**) and Suruga (**c**–**h**) bays near the inlet of the deep-sea water pumping stations at depths of 800 and 400 m, respectively. **a**: Eel swarm, including *Simenchelys parasitica* (i), Myxinidae (iii), and Synaphobranchidae (iii) (cast ID BCM3-9, November 17, 2020). **b**: *Centroscymnus coelolepis* (cast ID BCM3-9, November 17, 2020) **c**: *Pterothrissus gissu* (cast ID BCM4-2, April 17, 2021) **d**: *Dalatias licha* (cast ID BCM4-2, April 17, 2021) **e**: *Physiculus japonicus* (cast ID BCM2-5, February 16, 2020) **f**: *Hexanchus griseus* (cast ID BCM2-4, February 15, 2020) **g**: *Helicolenus hilgendorfii* (cast ID BCM2-5 February 16, 2020) **h**: *Coelorinchus* sp. (cast ID BCM2-4, February 15, 2020).

A total of 32 baited camera deployments were conducted during the same period as the eDNA sampling, including two deployments in Sagami Bay and 30 deployments in Suruga Bay, all of which were located near the inlets of the pumping stations (Supplementary Table S3). During each deployment, the video sequences were recorded for durations of 5.5–12 h in Sagami Bay and 2.5–12.5 h in Suruga Bay. In total, 17 h of video footage was recorded in Sagami Bay and 182 h in Suruga Bay using baited cameras (Supplementary Table S3). Using footage from the baited cameras, we identified the observed fish and other animals to the most specific taxonomic level possible, ranging from family to species (Fig. 8). Additionally, we estimated the population density of the detected animals by analysing their first arrival times. These identifications were compared with the results obtained from the eDNA analyses (Table 1 and Supplementary Table S4). In Sagami Bay, baited cameras recorded 11 fish taxa belonging to 7 families. Among them, five were identified at the species level, three at the genus level, and three at the family level. A comparison of with the eDNA analysis results revealed that two species corresponded at the species level and five to the family level. In Suruga Bay, baited cameras recorded 17 fish taxa belonging to 13 families, with 12 identified at the species level, 1 at the genus level, and 4 at the family level. Of them, the eDNA results corresponded to six fish at the species level and four fish at the family level. In addition, 10 invertebrates, including molluscs and arthropods, were recorded using baited cameras in Suruga Bay.

**Table 1:** Summary of animals recorded by baited camera observation. eDNA detection indicates the presence of the observed fish species, with “ +  + ”, “ + ”, “-” and “na” representing “same species identified by eDNA analysis”, “species from the same family identified by eDNA analysis”, “not identified by eDNA analysis”, and “not applicable for eDNA analysis”, respectively.

Site	Phylum	Family	Species	Number of occurrences in casts	%of occurrences in casts	Estimated population density No./km^2^	eDNA detection
**Sagami Bay**							
	Chordata	Myxinidae	unidentified	2	100	312,242.5	-
	Chordata	Somniosidae	*Centroscymnus coelolepis*	2	100	883.6	-
	Chordata	Somniosidae	*Centroscymnus owstonii*	1	50	187.9	+
	Chordata	Centrophoridae	unidentified	2	100	44.4	-
	Chordata	Centrophoridae	*Deania hystricosa*	2	100	16.6	+
	Chordata	Synaphobranchidae	unidentified	2	100	104,994.7	+
	Chordata	Synaphobranchidae	*Simenchelys parasitica*	2	100	17,128.2	+ +
	Chordata	Macrouridae	*Coryphaenoides* sp.	1	50	281.5	-
	Chordata	Macrouridae	*Coelorinchus* sp.	2	100	17.3	+
	Chordata	Anoplopomatidae	*Erilepis zonifer*	1	50	2.5	+ +
	Chordata	Psychrolutidae	*Ebinania* sp.	1	50	1.2	+
**Suruga Bay**							
	Chordata	Myxinidae	unidentified	11	36.7	652.2	-
	Chordata	Hexanchidae	*Hexanchus griseus*	3	10.0	23.9	+ +
	Chordata	Somniosidae	*Somniosus pacificus*	1	3.3	4.5	-
	Chordata	Somniosidae	*Centroscymnus owstonii*	7	23.3	52.7	-
	Chordata	Dalatiidae	*Dalatias licha*	20	66.7	120.6	+ +
	Chordata	Centrophoridae	unidentified	1	3.3	91.7	-
	Chordata	Centrophoridae	*Deania hystricosa*	5	16.7	71.9	+ +
	Chordata	Centrophoridae	*Centrophorus granulosus*	1	3.3	349.6	-
	Chordata	Albulidae	*Pterothrissus gissu*	27	90.0	2374.0	+ +
	Chordata	Synaphobranchidae	unidentified	29	96.7	23,981.6	+
	Chordata	Synaphobranchidae	*Simenchelys parasitica*	9	30.0	51.3	+ +
	Chordata	Congridae	*Congriscus megastomus*	5	16.7	3548.1	+
	Chordata	Moridae	*Physiculus japonicus*	26	86.7	2451.2	-
	Chordata	Macrouridae	*Coelorinchus* sp.	21	70.0	1207.7	+
	Chordata	Sebastidae	*Helicolenus hilgendorfii*	10	33.3	40.8	-
	Chordata	Gempylidae	*Promethichthys prometheus*	1	3.3	35.0	+ +
	Chordata	Trichiuridae	unidentified	1	3.3	91.6	+
	Mollusca	Cymatiidae	*Fusitriton oregonensis*	2	6.7	16.1	na
	Mollusca	Buccinidae	unidentified	6	20.0	68.8	na
	Mollusca	Buccinidae	*Buccinum leucostoma*	23	76.7	738.1	na
	Mollusca	Buccinidae	*Buccinum bombycinum*	1	3.3	2.1	na
	Arthropoda	Cirolanidae	*Bathynomus doederleinii*	24	80.0	120.2	na
	Arthropoda	Caridea	unidentified	12	40.0	1210.7	na
	Arthropoda	Pandalidae	*Pandalus nipponensis*	13	43.3	116.2	na
	Arthropoda	Pandalidae	*Heterocarpus* sp.	2	6.7	269.8	na
	Arthropoda	Nephropidae	*Metanephrops japonicus*	7	23.3	61.4	na
	Arthropoda	Inachidae	*Macrocheira kaempferi*	6	20.0	12.9	na

## Discussion

The aim of this study was to determine the effectiveness of eDNA metabarcoding and its potential to replace conventional, logistically and cost-intensive field survey methods for determining deep-sea fish biodiversity. To support the reliability of the results, baited cameras were used to observe different fish species, as well as other animals, around the inlets of pumping stations to compare with the eDNA metabarcoding data and provide a more comprehensive understanding of deep-sea fish biodiversity.

The pumped deep-sea water is transported from seabed intakes to pumping stations through long subsea pipelines and is supplied almost continuously, despite occasional interruptions. Although eDNA degradation during transport cannot be entirely excluded, the physical conditions within the pipeline system are likely to limit its impact, considering the transit time through the pipeline. In Sagami Bay, both collection methods detected similar numbers of fish species per sample, and the difference in sampling volume between the pumped and Niskin methods did not significantly affect the detected species richness, consistent with the accumulation curves for the two methods. Conversely, in Suruga Bay, a significant positive correlation in species detection using the Niskin method was observed, even when using a GLMM analysis that accounted for sampling period, consistent with the accumulation curves showing higher species detection with the Niskin method than with the pump method. Despite a larger volume difference in Sagami Bay, no significant difference was observed between the two methods. In contrast, Suruga Bay exhibited a significant difference despite a smaller volume gap (only 2 L). These contrasting results suggest that the difference observed in Suruga Bay is not primarily driven by the minor difference in filtration volume, but rather by the physical intake conditions, specifically earthquake-induced pipeline damage, which resulted in the intake lying directly on the seafloor. This condition may have altered the available species pool compared to that sampled by the Niskin method.

In Sagami Bay, all fish species detected by the pump method were consistent with those detected by the Niskin method. Furthermore, 15 fish species, including demersal fishes, such as *Allocareproctus jordani* and *Notacanthus chemnitzii,* were specifically detected using the pump method (Fig. 3, Supplementary Table S2-2, and Supplementary Fig. 2a and 3). This is likely due to the location of the pumping station inlet near the seafloor, which is more suited to the depth of these fish habitats. Conversely, in Suruga Bay, 28 fish species were specifically identified by the Niskin method, with occurrence rates ranging from 2.99% to 11.94%, corresponding to 2–8 filter samples; however, most fish species with over 50% occurrence rates were detected by both collection methods (Fig. 4, Supplementary Table S2-3, and Supplementary Figs. 2b and 4). Fish eDNA in the open ocean is typically identified at the depth at which it is released, or at greater depths; however, it is infrequently observed at shallower depths^46^. In both bays, the consistency between the habitat depth ranges of the detected fish species and the depths of pump station intakes indicated that eDNA sampling effectively reflected the vicinity of the inlet and the upper water column. Furthermore, no significant differences in the habitat depth distribution of the detected fish species (categorised as shallow, middle, and deep) between the collection methods indicated that both water collection methods successfully collected eDNA around the pumping inlets with similar fish compositions, regardless of habitat depth. These results indicate that deep-sea water from the pumping station was effective for eDNA sampling.

Hierarchical cluster analysis of eDNA samples collected using both methods in each bay showed that many samples were grouped together, with branches corresponding to the pump and Niskin methods often intermingled and forming closely related clusters. This pattern suggests a degree of overlap in the fish communities identified by the two water collection methods. NMDS analysis further supported this pattern, with samples from the two methods showing a degree of overlap in ordination space. The 95% confidence interval ellipsoids also showed considerable overlap, particularly in Suruga Bay. In Suruga Bay, multifactorial PERMANOVA revealed that the sampling period accounted for a significantly greater proportion of variation in community composition than the sampling method. Notably, when eDNA data within each sampling period were pooled, no significant differences in fish community composition were detected between the two methods. In contrast, as mentioned above, all fish species identified using the Niskin method in Sagami Bay were also detected using the pump method. Taken together, these results indicate that despite minor individual sample biases, the pump method provides a broadly comparable representation to the Niskin method for eDNA analysis of deep-sea fish biodiversity.

Using baited camera observations, 11 and 17 fish taxa were identified in Sagami and Suruga bays, respectively. Of the fish detected with baited cameras, 7 (in Sagami Bay) and 10 (in Suruga Bay) were also detected with eDNA methods. In Sagami Bay, four fish taxa were not detected by eDNA analysis, including three fish families (Myxinidae, Somniosidae, and Centrophoridae) and one genus, *Coryphaenoides* sp. In Suruga Bay, seven fish taxa were not detected using eDNA, including two families (Myxinidae and Centrophoridae) and five species: *Somniosus pacificus*, *Centroscymnus owstonii*, *Centrophorus granulosus*, *Physiculus japonicus*, and *Helicolenus hilgendorfii*. As baited camera observations are particularly effective at attracting predatory and scavenging species, most of the fish not detected by eDNA analysis were shark species, commonly considered top predators, with relatively low population densities and low detection probabilities in deep-sea environments^47–49^. Another reason for the lack of detection of these species using eDNA may be mismatches between the primers and templates^29^. Consequently, these species may be difficult to detect in the deep-sea using eDNA analysis. To improve the detection probability of rare eDNA, increasing sampling effort is generally needed. Although the pump method is technically capable of collecting larger volumes of water, our previous evaluation showed that increasing the filtration volume does not necessarily enhance detection efficiency^8^. Specifically, larger filtration volumes resulted in a significant decrease in the proportion of fish-target reads and an increased risk of PCR inhibition owing to the co-accumulation of inhibitory substances and non-target DNA^8^. Increasing the number of sample replicates, rather than filtration volume, has been suggested as a more effective approach to improve detection in eDNA analyses^8,50^.

Notably, among the species not detected by eDNA analysis, Myxinidae and *Physiculus japonicus* were found to have higher occurrence rates and higher estimated population densities using baited camera observations. Hagfish, which belong to the family Myxinidae and inhabit the ocean floor at depths over 100 m, are known as scavengers^51^. Baited camera observations have indicated that scavengers, including hagfish, are frequently among the first arriving animals when a baited camera is deployed on the seafloor^52^. Hagfish inhabit burrows in soft muddy seabed^53,54^. A recent study reported that fish species which live in sandy or muddy environments are rarely detected using eDNA^55^. These species tend to be relatively inactive and often hide in mud, which makes it more challenging for their DNA to be released or dispersed into the surroundings^55^. As described above, the primer sets used in this study had two and five primer/template mismatches for *Physiculus japonicus* and hagfish, respectively. Therefore, optimised primer sets are required to detect these fish species.

As previously reported, inconsistent detection between eDNA analysis and visual surveys, including baited camera observation, is a general characteristic of these methodologies^27,56,57^. One factor contributing to these inconsistencies is variability in the amount of eDNA released by different organisms^58^. Thus, combined analysis using eDNA and baited camera observations provides complementary detection of fish and represents an important survey method for understanding fish diversity^26,27^. By contrast, conducting deep-sea surveys using research vessels equipped with CTD water samplers and deploying baited cameras remains logistically demanding and time-consuming. Deep-sea water obtained from pumping stations is a practical and promising alternative; however, a limitation of this method is that the water intake is fixed at a single location and depth, potentially restricting the spatial representativeness of the detected fish community. This spatial limitation may be mitigated by the pump method’s ability to integrate eDNA from the surrounding water mass through continuous suction, compared with the single-point ‘snapshot’ provided by a Niskin bottle. In addition, Japan has several deep-sea water pumping stations located along different coastal regions, and the combined use of these facilities may further reduce the impact of this limitation. At the same time, as demonstrated by the results in Suruga Bay, where earthquake-induced pipeline damage likely affected species detection, it is important to confirm the actual physical condition and placement of the water intake when applying this monitoring approach to other facilities. The pumped method also facilitates sample collection at any time, making it highly suitable for continuous monitoring of deep-sea biodiversity, which is increasingly critical under ongoing climate change and human activities on marine ecosystems. This approach therefore has strong potential to support long-term ecological monitoring in coastal deep-sea areas.

## Supplementary Information

Supplementary Information 1.

Supplementary Information 2.

## Data Availability

Raw DNA sequence data from MiSeq were deposited in the DNA Data Bank of JAPAN (DDBJ) Sequence Read Archive (DRA; https://www.ddbj.nig.ac.jp/dra/index-e.html) under the BioProject number PRJDB18791. The external dataset used was map vector data (Natural Earth, www.naturalearthdata.com/).
