# Supplementary material for: Environmental DNA from pumped deep-sea water enables monitoring of deep-sea fish diversity
Source: Sci Rep. 2026 Jun 12;16:26870. doi: 10.1038/s41598-026-56998-1 (PMC13518824; doi:10.1038/s41598-026-56998-1)
Supplement: Supplementary file 1 [file 41598_2026_56998_MOESM2_ESM.docx]

**Environmental DNA from pumped deep-sea water enables monitoring of deep-sea fish diversity**

Takao Yoshida^1,2*^, Masaru Kawato^1,3^, Shinji Tsuchida^1,3^, Aya Yamazaki^1^, Yoshihiro Fujiwara^1,3^, Yuriko Nagano^1,2^, and Akinori Yabuki^1,2^

^1^Deep-Sea Biodiversity Research Group, Marine Biodiversity and Environmental Assessment Research Center, Research Institute for Global Change, Japan Agency for Marine-Earth Science and Technology (JAMSTEC), Yokosuka, Kanagawa, Japan

^2^ Institute for Extra-cutting-edge Science and Technology Avant-garde Research of Life (X-star), Japan Agency for Marine-Earth Science and Technology (JAMSTEC), Yokosuka, Kanagawa, Japan

^3^ Research Institute for Marine Technology and Engineering, Japan Agency for Marine-Earth Science and Technology (JAMSTEC), Yokosuka, Kanagawa, Japan

*** Corresponding author:**
Takao Yoshida
[tyoshida@jamstec.go.jp](mailto:tyoshida@jamstec.go.jp)

**Supplementary Information**

**Supplementary Method**

**Detailed DNA extraction, library preparation, and sequencing**

The process of DNA extraction, library preparation, and Sequencing were preformed following methods that have been previously reported. Environmental DNA (eDNA) was extracted from a filtration membrane within a Sterivex filter cartridge using the DNeasy Blood and Tissue Kit (QIAGEN, Valencia, CA, USA). The process involved opening the cartridge, removing the filter, and cutting it into small pieces to facilitate the lysis of eDNA sources. The eDNA was then eluted into 200 µl of elution buffer. The concentration of the extracted eDNA was measured using a Qubit dsDNA HS Assay Kit and a Qubit fluorometer (Life Technologies, Carlsbad, CA, USA).

To construct the library, a two-step PCR was performed for metabarcoding of fish in eDNA, following a modified method of a previously reported by Miya et al 2015. Extracted eDNA was used for multiplex PCR targeting the mitochondrial 12S rRNA gene sequence with two universal primer sets: 5'-ACACTCTTTCCCTACACGACGCTCTTCCGATCT-NNNNNN-GTCGGTAAAACTCGTGCCAGC-3’ (MiFish-U-Forward), 5'-ACACTCTTTCCCTACACGACGCTCTTCCGATCT-NNNNNN-GTTGGTAAATCTCGTGCCAGC-3’ (MiFish-E-Forward), 5'-GTGACTGGAGTTCAGACGTGTGCTCTTCCGATCT-NNNNNN-CATAGTGGGGTATCTAATCCC-3' (MiFish-U-Reverse), 5'-GTGACTGGAGTTCAGACGTGTGCTCTTCCGATCT-NNNNNN-CATAGTGGGGTATCTAATCCTAGTTTG-3' (MiFish-E-Reverse). The first PCR amplification was carried out using Platinum SuperFi II DNA polymerase (Thermo Fisher Scientific, Waltham, MA, USA) and 1 X SuperFi II buffer containing 200 µM of each dNTP, 0.6 µM of each primer, and a minimum of 40ng eDNA template. Based on the previous report (Miya et al., 2015), eight technical replicates were created for each eDNA template. PCR thermal cycling conditions were as follows; 98°C for 30 s for initial denaturation, followed by 38 cycles of denaturation at 98°C for 10 s, annealing at 60°C for 10 s, and extension at 72°C for 30 s; with a final extension at 72°C for 5 min. First PCR amplicons were analyzed by an E-gel Precast Agarose Electrophoresis System using an E-gel SizeSelect II Agarose Gels 2% (Thermo Fisher Scientific, Waltham, MA, USA). The second PCR and next-generation sequencing was performed by Bioengineering Lab. Co., Ltd (Sagamihara, Japan). First PCR products were purified with AMPure XP (Beckman Courter, Brea, CA, USA). Second PCR was performed to add unique index sequences and Illumina sequencing adapters (2ndF: 5'-AATGATACGGCGACCACCGAGATCTACAC-XXXXXXXX-ACACTCTTTCCCTACACGACGC-3', 2ndR: 5'-CAAGCAGAAGACGGCATACGAGAT-XXXXXXXX-GTGACTGGAGTTCAGACGTGTG-3') using Ex Taq polymerase (Takara Bio, Shiga, Japan). Adapter-attached PCR fragments were purified using AMPure XP, and then these paired-end sequence libraries were sequenced using Illumina MiSeq (Illumina, San Diego, CA, USA) under 2 × 300 bp conditions.

**Supplementary Fig. 1.** Map of Japan (a) and Sagami and Suruga Bays with sampling locations (b). The red section in (a) is magnified in (b). Red and blue circles represent the pumping stations in Sagami and Suruga Bays, respectively. Red and blue rectangles mark the inlets of these pumping stations in Sagami and Suruga Bays, respectively. The map was illustrated using RStudio with data from Natural Earth.

**Supplementary Fig. 2** Frequency of occurrence of ASVs detected among samples. Histograms of the occurrence rates of detected ASVs specific to the Niskin method (yellow), specific to the pump method (blue), and shared by both methods (light blue) are shown for samples from (a) Sagami Bay and (b) Suruga Bay. The Y- and X-axes indicate the number of ASVs and the occurrence ratio of each ASV among samples, respectively.

**Supplementary Fig. 3.** Composition of fish MOTUs in eDNA samples detected by two collection methods, the Niskin method and the pump method, in Sagami Bay. A heatmap based on sequence presence indicates the detected MOTU species for each water collection method: the Niskin method (yellow) and the pump method (blue). Pump method–specific MOTUs are represented by blue circles placed before the MOTU names, and the remaining MOTUs are shared between the Niskin and pump methods. MOTU order and eDNA sample order were defined by clustering using the unweighted pair group method with arithmetic mean and the Jaccard index.

**Supplementary Fig. 4.** Composition of fish MOTUs in eDNA samples detected by two collection methods, the Niskin method and the pump method, in Suruga Bay. A heatmap based on sequence presence indicates the detected MOTU species for each water collection method: the Niskin method (yellow) and the pump method (blue). Niskin method-specific MOTUs and pump method-specific MOTUs are represented by yellow and blue circles, respectively, placed before the MOTU names. The remaining MOTUs are shared between the Niskin and pump methods. MOTU order and eDNA sample order were defined by clustering using the unweighted pair group method with arithmetic mean and the Jaccard index.

**Supplementary Data**

**The constructed ASV sequences in FASTA format are shown below**

>Apristurus_cf._macrorhynchus

CGGTTATACGAGTAACCCACATTAATACTTCCCGGCGTAAAGAGTGGTTTAAGGACGGCC

TATTACAACTAAAGTTAAGACCCCATCATGCTGTTATACGCACTTATGGGTGGAATAAAC

AACAACGAAAGTGACTTTATAAAAAAAGGGGCCTTGATGCCACGATATTTGAA

>Prionace_glauca

GCGGTTATACGAGTAACTCACATTAATACACCCCGGCGTAAAGAGTGATTAAAGAATGAC

CTTTAACTACTAAAGTTTAGACCTTATTAAACTGTTATACGTATTCATAAATGGAATAAA

CAACAACGAAAGTGACTTTACGAATTAAGGAACCTTGATGTCACGACAGTTGG

>Sphyrnidae_sp.

CGGTTATACGAGTGACTCACATTAACACACCACGGCGTAAAGAGTGATTAAAGAATGACC

TCAAACTTACTAAAGTTCAGACCTCATAAAGCCGTTATACGCATCCATGAGTAGAATAAA

CAACAACGAAAGTGACTTTATAAATATAAGAAACCTTGATGTCACGACAGTTGG

>Chlamydoselachus_anguineus

CGGTTATACGAGTAACCCAAATTAATACTTTTCCGGCGTAAAGAGTGGTTAAAGAAAAAA

ACAACTAAAGTTAAGACACCATCAAGCTGTTATACGCATTTATGAGTGGAATAACCAATA

ACGAAAGTGACTTTATAATATTAAAATCTTGATGCCACGACAGTTA

>Hexanchus_griseus

GCGGTTATACGAGTGACCCAAATTAATATTTCCCGGCGTAAAGAGTGGTTTAAGAATAAA

TCTCAAATAATTAAAGTTAAGATATCATCAAACTGTTATACGTATTTATGAATAAAATAA

TCAACTACGAAAGTAACTTTATAAAATACTAGAACTCTTGATGCCACGACAGGTA

>Squalidae_sp.

GCGGTTATACGAGTGACCCTTATTAATATTTTCCCGGCGTAAAGAGTGGTTTAAGAAAAT

CTTAAACAACTAAAGTTAAGACCTCATCAAGCCGTTATACGCTCTCATGAAAAGAATTAT

CAACAACGAAAGTGACTTTATAATAATAGAGACCTTGATGCCACGACAGTTG

>Deania_sp.

GCGGTTATACGAGTAACCCTTATTAATACTTCCCCGGCGTAAAGAGTGGTTTAAGAAAAA

TCCTTTAAATAATTAAAGTTAAGACCTCATCAAGCTGTTATACGCCCCCATGATTGGAAC

AATCGACGACGAAAGTGACTTTATAATATTAGAAATCTTGATGCCACGACAGTTGG

>Deania_hystricosa

GCGGTTATACGAGTAACCCTTATTAATACTTCCCCGGCGTAAAGAGTGGTTTAAGAAAAA

TCCTTTAAATAATTAAAGTTAAGACCTCATCAAGCTGTTATACGCCCCTATGATTGGAAC

AATCAACGACGAAAGTGACTTTATAATATTAGAAATCTTGATGCCACGACAGTTG

>Centroscymnus_owstonii

CGGTTATACGAGTAACCCTTATTAATACTTCCACGGCGTAAAGAGTGGTTAAAAAAATCT

TAAATAACTAAAGTTAAGACCTTATCAAGCTGTTATACGCTCTCATAAGTAGAATTATCG

ACAACGAAAGTGACTTTATAATATTAGAAATCTTGACTCCACGACAGTTG

>Etmopteridae_sp.1

CGGTTATACGAGTGACCCTAATTAATATTTACCCGGCGTAAAGGGTGGTTTAAGGAAAAA

TCTTTAAAGATTAAAGTTGAGATCCTATTAAGCTGTTATACGCTCTCATAGGAGGAATTA

TCAATAACGAAAGTAACTTTATTAAATTAGATAACTTGATGCCACGATAGTTG

>Zameus_ichiharai

GCGGTTATACGAGTAACCCTTATTAATATTTTTACGGCGTAAAGAGTGGTTTAAAAAAAT

CTTAAATAACTAAAGTTAAGACCTTATCAAGCTGTTATACGCTCTCATAAGTAGAATTAT

CGACAACGAAAGTGACTTTATTATACTAGAAATCTTGATCCCACGATAGTTG

>Dalatias_licha

CGGTTATACGAGTAACCCTTATTAATATTCCCTCGGCGTAAAGAGTGGTTTAAGAAAATC

TTAACTAACTAAAGTTATGACCTCATCAAGCTGTTATACGCTCCTATGACTGGAATTATC

AACAACGAAAGTGACTTTACAACATCAGAAACCTTGATGCCACGACAGTTG

>U98.5_Dalatias_licha_gotu1

GCGGTTATACGAGTGACCCTTATTAATATTCCCTCGGCGTAAAGAGTGGTTTAAGAAAAT

CTTAACTAACTAAAGTTATGACCTCATCAAGCTGTTATACGCTCCTATGGGTGGAATTAT

CAACAACGAAAGTGACTTTACAACATCAGAAACCTTGATGCCACGACAGTTG

>Pterothrissus_gissu

GCGGTTATACGAGAGGCTCAAATTGATGCTCGTCGGCGTAAAGAGTGATTACAGGAATAT

AAACAACTAAAGCCGAATACCCCTCAGGCTGTTATACGCATCTGGGGAAATGAAGCCCCA

AAACGAAGGTAGCTTTAATATATTAACCTCGAATTCACGACAACTG

>Notacanthus_abbotti

GCGGTTATACGAGAGGCTCAAATTGATGATTAACGGCGTAAAGAGTGATTAAAGGAGTAA

GACAACTAAAGCCGAACCCCTCTTAGGCCGTCATACGCTTCTAGAGATGTGAAGCCCCAA

AACGAAAGTAGCTTTAACCCAACCCCTTGAACTCACGACAGCTAA

>Notacanthus_chemnitzii

CGCGGTTATACGAGAGGCTCAAATTGATGATCAACGGCGTAAAGAGTGATTAAAGGAGTA

AAATAACTAAAGCCGTACCCCTCTTAGGCCGTCATACGCTTCTAGAGATGTGAAGCCCCA

AAACGAAAGTAGCTTTAATTTAACCCCTCGAATTCACGACAGCTA

>Simenchelys_parasitica

CGGTTAGACGAGAGGCTCAAATTGACACTCTTTCGGCGTAAAGCGTGATTAGAGACCACA

ACACACTAAAGCCAAACACCTCCTAAGCTGTCATACGCAACCAGAGGCAGGAAGCCCCCC

AACGAAAGTAGCTTTAATAAACATCTTGAATTCACGACAGTTA

>Synaphobranchidae_sp._1

GCGGTTATACGAGGGGCTCAAGTTGACACTTTTTCGGCGTAAAGCGTGATTAAGGACTAT

AATAAACTAAAGCCAAACACCTCCCAAGCTGTCATACGCAATCGGAGGCAGGAGGCCCCC

CAACGAAAGTAGCTTTAATAAACAAATCTTGAATTCACGACAGTTA

>U98.5_Synaphobranchidae_sp._1

CGGTTATACGAGGGGCTCAAGTTGACACCTTTTCGGCGTAAAGCGTGATTAAAGACTATA

ATAAACTAAAGCCAAACACCTCCCAAGCTGTCATACGCAATCGGAGGCAGGAGGCCCCCC

AACGAAAGTAGCTTTAATAAACAAATCTTGAACTCACGACAGTTAA

>Neenchelys_mccoskeri

GCGGTTATACGGGGGGCCCAAACTGATATTCAACGGCGTAAAGCGTGATTAAGGAAAATT

AAAAACTAAAGAAGAAGGTCCCCCAAGCTGTTATACGCTAACGGGATGACTGAACCCCCA

CAACGAAAGTGACTTTAAAACTCTTGAACCCACGAATATTA

>U98.5_Ophichthus_serpentinus_gotu1

CGCGGTTATACGAGAGGCTCAAATTGACATTCTACGGCGTAAAGCGTGATTAGAGAAAAC

AAAAACTAAAGCCGAACATCTCCTTAACTGTTATACGTTCAAAGAGACATGAAGCCCCAC

AACGAAAGTAGCTTTAACCCAAAATCTTGAACTCACGAAAATTA

>Derichthyidae_sp.

GCGGTTATACGAGGGGCTCAAATTGATATCCTTCGGCGTAAAGTGTGATTAGAGAACAAT

AAAAACTAAAGCTAAACACCTCCTGAGCTGTCATACGCTAACGGAGACAGAAACCCCACA

ACGAAAGTAGCTTTAACAACTCTTGAACTCACGACCATTA

>Muraenesox_cinereus

GCGGTTAGACGACGAGGCTCAAATTGATGTTCCACCGGCGTAAAGCGTGATTAGATAAAT

TAAAAAACTAAAGCCAAATACACCCATAGCTGTTATACGCTTACGGACTACTAGGCCCCA

CCACGAAAGTGGCTTTAACACACTATTGAATTCACGACCGCTA

>Acromycter_nezumi

CGGTTATACGAGGGGCTCAAATTGATATTCTCCGGCGTAAAGAGTGATTAAAGAAATTAA

CTAAACTAAAGCCAAATACCTCCTAAGCTGTCATACGCTAATGAAGATACGAAGCCCCAC

AACGAAAGTGGCCTTATTACACTTGAACTCACGACCATTA

>Congriscus_sp.

CGGTTATACGAGAGGCTCAAATTGATATCCTTCGGCGTAAAGTGTGACTAGAGAAAATAA

TGAAACTAAAGCCAAACATCTCCCCAGCTGTCATACGCTAACGGAGCCAGAAGCCCCACA

ACGAAAGTGGCTTTAATAATTCTTGAACTCACGACCATTAAG

>Gavialiceps_taeniola

GCGGTTATACGAGAGGCTCAAATTGATACTCTCCGGCGTAAAGAGTGATTAAAGAGAAGA

CAAAACTAAGGCCAAACATCTCCTAAGCTGTCATACGCTGATGAAGACATGAAGCCCCAC

AACGAAGGTGGCCCTAAAACCCTTGAACTCACGACCATTA

>Nettastoma_parviceps

CGGTTATACGAGAGGCTCAAATTGATAATCCACGGCGTAAAGCGTGATTAAAGAAAAAGT

AAAACTAAGGCCAAACACCCCCCAAGCTGTCATACGCTAACGGACACACGAAGCCCGAAA

ACGAAAGTGGCCCTAAAACCCTTGAATTCACGACCATTA

>U98.5_Nettastomatidae_sp.

GCGGTTATACGAGAGGCTCAAATTGATATTCTTCGGCGTAAAGCGTGATTAGAGGAAAAA

CAAAACTAAAGCCAAACATCCCCCAAGCTGTCATACGCTAACGGAGAAACGAAGCCCCAC

GACGAAGGTGGCCTTAAAAACCTTGAACTCACGACCATTA

>Engraulis_japonicus

CGGTTATACGAGAGACCCTAGTTGATTGAAGCGGCGTAAAGAGTGGTTATGGAATTTTCT

ACCCTAAAGCAGAAAACCTCTCAAACTGTTATACGCACCCAGAGGTTGAAACCCCTTACA

CGAAAGTGACTTTATTTTCGCCTACCAGAAGCCACGAAAGCTA

>Etrumeus_teres

CGGTTATACGAGAGGCCCTAGTTGACACATTCCGGCGTAAAGAGTGGTTATGGGAATTAA

AAACTAGAGCTGAAGACTCCTAGGGCCGTTATACGTTTTCCAGGAGTTCGAAACACTAAA

ACGAAAGTAGCTCTATCCTTACCTACCAGACTCCACGACAGCTGA

>Sardinella_zunasi

GCGGTTATACGAGAGGCTCGAGTTGATAATCTCGGCGTAAAGAGTGGTTATGGAGAAGAC

TAAACTAAAGCTGAAGACCCCCCAGGCTGTTTAACGCATGCGGGTGTTCGAACCACTTAT

ACGAAAGTAGCTTTAACGCATTCCACCAGAATCCACGACAGCTGGG

>Sardinops_sp.

CGCGGTTATACGAGGGACCCTAGTTGATTTAATCGGCGTAAAGAGTGGTTATGGAGAATA

AGAAACTAAAGCCGAAGACCTCTTAGGCCGTCATACGTACCTAGAGGCTCGAATAACAAA

CACGAAAGTAGCTTTACCCCTTCCTGCCAGAACCCACGAGAGCTG

>Gonorynchus_abbreviatus

CGGTTATACGAGAGGCTCCAGTTGACAGTCACGGCGTAAAGAGTGATTAAGGCGAATCTT

GAACTAAGGCCGAAGACCCCCTCAGCCGTTATACGCGTTTTGGAGGTGTTAAGCTCACCC

ACGAAAGTGGCTTTACCATGAACCACCTGACTTCACGATAGCCG

>Bathylagus_pacificus

CGGTTATACGAGAGGCCCAAGTTGATGATCACCGGCGTAAAGAGTGGTTACGGAAATACT

TCACTAAAGCCGAACACCCTCCAGGCTGTCATACGCATCTGAGGCTACGAAGCTCTCCTA

CGAAAGTGGCTTTACTCCTCCCTGAACCCACGACAGCTA

>Leuroglossus_schmidti

GCGGTTATACGAGAGGCCCAAGTTGACAACCACCGGCGTAAAGAGTGGTTATGGAAACAC

TATACTAAAGCCGAACACCCTCTAGGCTGTCATACGCACCTGAGACTACGAAGCTCCCCC

ACGAAAGTGGCTTTATTCCTCCCTGAACCCACGACAGCTACG

>Lipolagus_ochotensis

CGGTTATACGAGAGGCCCAAGTTGATGACCACCGGCGTAAAGAGTGGTTACGGAAGCACT

TCACTAAAGCCGAACAACCTCTAGGCTGTCATACGCACCTGAGGCTACGAAGCTCCCCCA

CGAAAGTGGCTTTACCCCTCCCTGAACCCACGACAGCTA

>Pseudobathylagus_milleri

GCGGTTATACGAGAGGCCCAAGTTGACAATCACCGGCGTAAAGCGTGGTTATGGAACTTG

TAAAACTAAAGCCGAACACCCTCTAGGCTGTTATACGCATCTGAGACTACGAAGCTCCCC

TACGAAAGTGACTTTATCCCTCCTGAACCCACGACAGCTA

>U98.5_Bathylagus_pacificus_gotu1

GCGGTTATACGAGAGGCCCAAGTTGATAATCACCGGCGTAAAGAGTGGTTACGGAAATAT

TTCACTAAAGCCGAACACCCTCTAGGCTGTCATACGCATCTGAGGCTACGAAGCTCTCCT

ACGAAAGTGGCTTTACCCCTCCCTGAACCCACGACAGCTA

>Alepocephalus_umbriceps

CGGTTATACGAGAGGCCCCAGTTGATAGGTGCGGCGTAAAGAGTGGTTATGGAGTACACC

CAACTAAAGCCGAAGACCCCCTAGGCTGTCATACGCACCTGGGGGCACGAAGCCCAATTA

CGAAAGTAGCTTTACTCAAGCCCACCCGACCCCACGACAGCTG

>Leptoderma_sp.

GCGGTTATACGAGAGGCCCCAGTTGATAAGTACGGCGTAAAGAGTGGTTATGGAGTGCAC

CCGACTAAAGCCGAAGACCCCCTCGGCCGTCATACGCATCTGGGGGCACGAAGCCCTATC

ACGAAAGTAGCTTTATTTAAGCCCACCCGACCCCACGACAGCTG

>Leptoderma_retropinna

GCGGTTATACGAGAGACCCCAGTTGATAAGTACGGCGTAAAGAGTGGTTATGGAGTGCAC

CCGACTAAAGCCGAAGACCCCCTCGGCCGTCATACGCATCTGGGGGCACGAAACCCTATC

ACGAAAGTAGCTTTATTTAAGCCCACCCGACCCCACGACAGCTGAGA

>Talismania_antillarum

GCGGTTATACGAGAGGCCCCAGTTGACGGGTACGGCGTAAAGAGTGGTTAAGAAAGAATC

CGACTAAAGTCGAAGACCCCTTAGGCCGTCATACGCACCTGGGGGCACGAAGCCCCGCCA

CGAAAGTAGCTTTACTAAAGCCCATCTGACCCCACGATAGCTG

>U98.5_Alepocephalidae_sp.

GCGGTTATACGAGAGGCCCCAGTTGATACATACGGCATAAAGAGTGGTTATGGAGTGTAC

CCAACTAAAGCTGAAGACCCCCTTGGCCGTTATACGCACCTGGAGGCACGAAGCCCCATC

ACGAAAGTAGCTTTACCTAAACCCACCCGACCCCACGACAACTGA

>U98.5_Rouleina_maderensis_gotu1

GCGGTTATACGAGAGGCCCTAGCTGATATTTACGGCGTAAAGAGTGGTTATGGAACACAC

ACAACTAAAGCTGAAGTCCCCCTCCGCCGTCATACGCACCTGGGGGCACGAAACCCTATT

ACGAAAGTAGCTTTACTCAAACCTGCCCGACCCCACGATAACTG

>Sigmops_elongatus

CGGTTATACGAGGGACCCAAATTGATAGCTACCGGCGTAAAGCATGATTGGGGGTACAAC

CAACTAAAGCCCAACCCACCCCCGCTGTCATACGCCTCCGGAAAAATGAAAACCCACCAC

GAAAGTAGCTTTAACCACACCTGAACTCATGACAACTA

>Sigmops_gracilis

CGCGGTTATACGAAAGACCCAAGTTGATAATCACCGGCGCAAAGCATGGTTAAGGACATA

ATAAACTGAAGCTGAACCCTCCCCCTGCTGTTATACGCCCCCGGAAAAATGTACCCCTAT

CACGAAAGTAGCTTCATCCCCACCTGAACCCATGACATCTA

>Maurolicus_japonicus

CCGCGGTTAGACGAGAGACCCAAGTTGACATCAAACGGCGTAAAGAGTGGTTAAGAACCT

TCCAAACTAAAGCTAAACCCCCTCCTGGCCGTTATACGCATCCGAAAGGGTGAGACACCA

CTACAAAAGTAGCTTTATAATTGCCTGACTCCACGACAGTTA

>Maurolicus_sp.

GCGGTTAGACGAGAGACCCAAGTTGACATCAAACGGCGTAAAGAGTGGTTAAGAACCTTC

CAAACTAAAGCTAAACCCCCTCCTGGCCGTTATACGCATCCGAAAGGGTGAGACACCACT

ACAAAAGTAGCTTTATAATTACCTGACTCCACGACAGTTA

>Polyipnus_matsubarai

CGGTTATACGAGAGGCCCAAGTTAACAGCCTCCGGCGTAAAGCGTGGACAAGGGTAAAAA

CAACTAAAGCCGAACGTCCTCTTAGCAGTTATACGCATTCGAAGGAACGAAGCCCTACCA

CGAAAGTGGCTTTATTTCACCTGATTCCACGACAGCTA

>Ichthyococcus_elongatus

GCGGTTATACGAGAGGCCCAAGTTGACAGACATCGGCGTAAAGAGTGGTCAAGGGTAGAA

CTAACTAAAGCCGAACGTCCCCCTGGCTGTTATACGCACTCGGAGGAATGAAGCCCCACC

ACGAAAGTGGCTTTACCCCACCTGAATCCACGACAACTAAG

>Polymetme_elongata

GCGGTTATACAAGAGGCCCAAGTTGATAGACGCCGGCGTAAAGAGTGGTCAGGGATACAA

TCAACTAAGGCCGAACATCCCCCTGGCTGTTATACGCATTCGGAGGAATGAAGCCCTACC

ACGAAAGTGGCTTTATCTTTGCCTGAACCCACGACAGCTA

>Astronesthes_cyanea

GCGGTTATACGAGAGGCCCAAGTTGACAGACACCGGCGTAAAGAGTGGTTAAGGATTAGA

CTTACTAAAGCCGAACGTCCCCCTGGCTGTTATACGCACTCGGAGGAATGAAGCCCCACT

ACGAAAGTGGCTTTATCATACCTGAACCCACGACAACTA

>Stomiidae_sp.1

CGGTTATACGAGAGACCCAAGTTGACAGGTGCCGGCGTAAAGGGTGGTTAAGGACAAAAA

TTACTAAAGCCGAACATCCCCTTGGCTGTTATACGCACCCGGAGGATTGAAGCCCCACCG

CGAAAGCGGCTTTAATTTGCCTGAACCCACGACAGCTA

>Astronesthes_indicus

GCGGTTATACGAGAGGCCCAAGTTGACAGATACCGGCGTAAAGAGTGGTTAAGGGTACAA

CTCACTAAAGCCGAACGTCCCCCTGGCTGTTATACGCACTCGGAGGAATGAAGCCCCACT

ACGAAAGTGGCTTTACCACACCTGAACCCACGACAACTA

>Stomiidae_sp.2

CGGTTATACGAGAGGCCCAAGTTGACAGACACCGGCGTAAAGAGTGGTCAAGGGTAAGAA

CAACTAAAGCCGAACATCCCCCTAGCTGTTATACGCACTCGGAGGAATGAAGCCCCACCA

CGAAAGTGGCTTTACTTCACCTGAACCCACGACAACTA

>Stomias_affinis

CGGTTATACGAGAGGCCCAAGTTGACAGACACCGGCGTAAAGAGTGGTCAAGGGTGAAAA

CAACTAAAGCCGAACGTCCCCCTGGCTGTTATACGCACTCGGAGGAATGAAGCCCCACCA

CGAAAGTGGCTTTACCCTACCTGAACCCACGACAACTA

>U98.5_Stomiidae_sp.

GCGGTTACACGAGAGGCCCAAGTTGACAATTACCGGCGTAAAGAGTGGTTAAGGGCAAAA

TACTAAAGCCGAACGTCCCCCTGGCTGTTATAAGCACTCGGAGGAAGGAAGCCCCACCAC

GAAAGTAGCTTTACCTTTCCTGAACCCACGACAGCTA

>U98.5_Stomias_affinis_gotu1

CGCGGTTATACGAGAGGCCCAAGTTGACAGACAACGGCGTAAAGAGTGGTCAGGCGTGAA

AACAACTAAAGCCGAACGTCCCCCTGGCTGTTATACGCACTCGGAGGAACGAAGCCCCAC

CACGAAAGTGGCTTTACTCAACCTGAGCCCACGACAACTAAGA

>Harpadon_microchir

CGCGGTTATACGAGAGGCCCGAGTTGACAAACATCGGCGTAAAGTGTGGTTAGGACTTTT

CCCACCTAAAGTTAAACACCCCCAGAACTGTTATACGCTCCCGGGAGTAGGAGGCCCAAC

AACGAAAGTGACTTTAAATATCCGACCCCACGACAGCTA

>Saurida_microlepis

CGGTTATACGAGAGGCCCGAGTTGATAAACACCGGCGTAAAGTGTGGTTAGGAATTTTCC

CTCTAAAGTAAAACACCCCCAGAACTGTTATACGCTCCCGGGGGCAGGAAGCCCAACAAC

GAAAGTGACTTTAAACCTCCGACTCCACGACAGCTACGA

>Lestrolepis_intermedia

GCGGTTATACGGGGGACCCAAGCTGATAGTCACCGGCGTAAAGCGTGGTTAAGGAACCAA

TCAACTAAAGTCAAACATTTCCTTGGCTGTTATAAGCCCCCGGAAGTTGGAAACCCCTCT

ACGAAAGTGACTTTAATACCCCTCCCGAATCCACCACAGCTAG

>Neoscopelus_microchir

CGCGGTTATACGAGAGGCCCAAGCTGATAGGAGACGGCGTAAAGAGTGGTTAGGGAAATT

CTTATACTAAAGCTAAACGCCTACAAAGCCGTTATACGCACCCGAAGGCATGAAAACCCC

CCACGAAAGTGGCTTTAACCCCACCCCGAACCCACGACAGCTT

>U98.5_Neoscopelidae_sp.

GCGGTTATACGAGAGGCCCAAGCTGATAGGAAACGGCGTAAAGAGTGGTTAGGGAGACCT

AGTACTAAAGCTAAACGCCTACAGAGCCGTTATACGCACCCGAAGGCATGAAAACCCCCC

ACGAAAGTGGCTTTAACCCCACCCCGAACCCACGACAGCTT

>Benthosema_pterotum

GCGGCCATACGAGTGTGCCCAAGCGGACAGTCTGTCGGCGTAAAGCGTGGTTAGGGATAA

ATTATTAACTACTAAAGCTAAACCCCATCAAGGCCGTAATACGCACCCGATGATGTGAGA

CCCAACCACGAAGGTGGCTTTAAACAAACCCGAACCCACGAGAGCCG

>Bolinichthys_distofax

CGGTCATACGAGTGTTAGCCCAAGCAGATGATCAACGGCGTAAAGAGTGGTTAGGGGCCC

TTGCAACTAAAGCTGAACGCCCTCCGGGCCGTTATACGCATCCGACGGTATGAAACCCCA

CCACGAAAGTGGCTTTAACCCAACCCGAACCCACGACAGCTA

>Diaphus_chrysorhynchus

GCGGTCAAACGAGGTAGAGCCCAAGCGGATAACCAACGGCGTAAAGAGTGGTTAGGGAAA

CCCACAACTAAAGCTGAATGCCCACAGAGCCGTGATACGCATCCGATGGTATGAAACCCC

ACCACGAAAGTGGCTTTAACCCAACCCGAACCCACGACAGCTAGG

>Diaphus_garmani

CGGTCAAACGAGGTTGTGCCCGAGCGGATGACCAACGGCGTAAAGCGTGGTTAGGGAAAC

CCTCTACTAAAGCTGAATACCCACAGAGCCGTGATACGCATCCGACGGCATGAAACCCCA

CCACGAAAGTGGCTTTAATCCAACCCGAACCCACGACAGCTAGGA

>Myctophidae_sp.1

GCGGTCAAACGAGGTAGTGCCCAAGCGGATAACCAACGGCGTAAAGAGTGGTTAGGGAAA

CCCACAACTAAAGCTGAATGCCCACAGAGCCGTGATACGCATCCGATGGCATGAAACCCC

ACCACGAAAGTGACTTTAACCCAACCCGAACCCACGACAGCTAG

>Diaphus_sp._NMMBP1206

GCGGTCAAACGAGGTTGTGCCCGAGCGGATGACCAACGGCGTAAAGCGTGGTTAAGGAAG

TCCCCTACTAAAGCTGAATACCCACAGAGCCGTGATACGCATCCGACGGCATGAAACCCC

ACCACGAAAGTGACTTTAATTCAACCTGAACCCACGACAGCTA

>Diaphus_suborbitalis

GCGGTCAAACGAGGTTGTGCCCGAGCAGACGACCAACGGCGTAAAGAGTGGTTAGGGAAG

CCCACAACTAAAGCTGAATGCCCACAAAGCCGTGATACGCATCCGATGGTATGAAACCCC

ACCACGAAAGTGACTTTAACCCAACCCGAACCCACGACAGCTA

>Myctophidae_sp.2

GCGGTCAAACGAGATTGTGCCCAAGCGGATAGCCAACGGCGTAAAGAGTGGTTAGGGAGA

CCCCCCAACTAAAGCTGAACACCCACAGAGCCGTGATACGCATCCGACGGCATGAAACCC

CACCACGAAAGTGGCTTTAACCCAGCCCGAACCCACGACAGCTAG

>Lampadena_yaquinae

GCGGCCATACGAGTGTTAGCCCCAGCGGATGGTCAGCGGCGTAAAGAGTGGTTAGGGGCC

CTTGCAACTAAAGCTAAATGTCCTCAAGGCTGTTATACGCATCCGACGACGTGAAACCCC

GCCACGAAAGTGGCTTTAACCCAACCCGAACCCACGGCAACTA

>Lampanyctus_alatus

GCGGTCATACGAGTGTTAGCCCAAGTGGATGGTCAACGGCGTAAAGAGTGGTTAGGGGCC

TCCAAAACTAAAGCTGAACGCCCTCCGGGCTGTTATACGCATCCGAGAGCATGAAACCCT

GCCACGAAGGTGGCTTTAACCTTACCCGAACCCACGACAGCTA

>Lampanyctus_jordani

CGCGGTCATACGAGTGTTAGCCCAAGTGGATAGTCAACGGCGTAAAGAGTGGTTAGGGAA

ACCCAAAACTAAAGCTGAACGCCCACAGAGCCGTTATACGCATCCGAGAGCATGAAACCC

CACCACGAAGGTGGCTTTAACCTTACCCGAACCCAGGACAGCTA

>Myctophidae_sp.3

CGGTCATACGAGTGTTAGCCCAAGTGGATGGTCAACGGCGTAAAGAGTGGTTAGGGACCC

CCTAAAACTAAAGCTGAACGCCCGCAGGGCCGTTATACGCACCCGAGAGCATGAAACCCC

ACCACGAAGGTGGCTTTAACCTAACCCGAACCCACGAAAGCTA

>Myctophidae_sp.4

GCGGTCATACGAGTGCGCCCAAGTGGACAGCCAACGGCGTAAAGTGTGGTTAGGGACCCA

ACAAACAACTAAAGCAGAACCACCTCCCGGCCGTCATACGCTTCTGACAGCGTGAAACCC

TCCCACGAAAGTGGCTTTAACCTACCCGAACCCACGAGAGCTA

>Myctophum_brachygnathum

CGGTCATACGAGTGTAGCCCAAGTGGACAGCTAGCGGCGTAAAGTGTGGTTAGGGGCCAC

TTAATACTAAAGCAGAACCACCTCCCGGCTGTTATACGCACCCGAAAGCGTGAAGCCCTC

CCACGAAAGTGACTTTAACTTCCCCCGAACCCACGAAAGCCA

>Myctophum_orientale

GCGGTCATACGAGTGTCGCCCAAGTGGACAGCTGGCGGCGTAAAGTGTGGTTAGGGGCCT

AAACAAGTACTAAAGCAGAACCACCTCCCGGCCGTTATACGCACCCGAAAGCGTGAAGCC

CTACCACGAAAGTGACTTTAACCCCACCCGAACCCACGAAAGCTA

>Myctophidae_sp.5

GCGGTCATACGAGTGTTAGCCCAAGTGGATGGTCAACGGCGTAAAGAGTGGTTAGGGAAT

CCCAGAACTAAAGCTGAACGCCCACAGGGCCGTTATACGCATCCGAGAGCATGAAACCCC

GCCACGAAGGTGGCTTTAACCTTACCCGAACCCACGACAGCTAGG

>Myctophidae_sp.6

GCGGTCATACGAGTGTTAGCCCAAGTGGATGGTCAACGGCGTAAAGTGTGGTTAGGGAAG

CCCAATACTAAAGCTGAACGCCCACAGGGCCGTTATACGCATCCGAGAGCATGAAACCCC

GCCACGAAGGTGGCTTTAACCTTACCCGAACCCACGACAGCTA

>Myctophidae_sp.7

GCGGTCATACGAGTGTAAGCCCAAGTGGATGGTCAACGGCGTAAAGTGTGGTTAGGGAAG

CCCAATACTAAAGCTGAACGCCCACAGGGCCGTTATACGCATCCGAGAGCATGAAACCCC

GCCACGAAGGTGGCTTTAACCTTACCCGAACCCACGACAGCTA

>Notoscopelus_sp._HUMZ_220402

GCGGTCATACGAGTGTTAGCCCGAGCAGATAGTCAACGGCGTAAAGAGTGGTTAGGGGTA

CTATATAACTAAAGCTGAACGCTCACAAGGCCGTTATACGCATCTGAGAGCATGAAACTC

CCCCACGAAAGTGGCTTTAACCCAACCCGAACCCACGACAGCTAGGA

>Notoscopelus_sp._HUMZ_222547

CGGTCATACGAGTGTTAGCCCGAGCAGATAGTCAACGGCGTAAAGAGTGGTTAGGGATAC

TATATAACTAAAGCTGAACGCTCGCAAGGCCGTTATACGCATCTGAGAGCATGAAACTCC

CCCACGAAAGTGGCTTTAACCCAACCCGAACCCACGACAGCTA

>Stenobrachius_sp.

CGCGGTCATACGAGTGTTAGCCCAAGCAGATGGTCAACGGCGTAAAGAGTGGTTAGGGAA

CCCTAAAACTAAAGCTGAACGCCCGCAGGGCCGTTATACGCATCCGACAGCATGAAACCC

CACCACGAAAGTGGCTTTAACCTGACCCGAACCCACGACAGCTA

>U98.5_Myctophidae_sp.1

GCGGCCATACGAGTGTTAGCCCCAGCGGATGGTCAGCGGCGTAAAGAGTGGTTAGGGACC

CTTACAACTAAAGCTAAATGTCCTCAAGGCTGTTATACGCATCCGACGGCATGAAACCCC

GCCACGAAGGTGGCTTTAACCCGACCCGAACCCACGACAACTA

>U98.5_Myctophidae_sp.2

CGGTCATACGAGTGTTAGCCCAAGTGGATGGTCAACGGCGTAAAGAGTGGTTAGGGGCCC

CCCCTAAAACTAAAGCTGAACGCCCGCAGGGCCGTTATACGCACCCGAGAGCATGAAACC

CCACCACGAAGGTGGCTTTAACCTGACCCGAACCCACGAAAGCTA

>Trachipterus_ishikawae

GCGGTTATACGAGGGGCCCAAGTCAATAGCCCCCGGCGTAAAGGGTGGTTAGGATTTTCC

CCAAAAACTAAGGCCCAACATTCTCAAGGCTGTTATACGCACCCGAGAAATGGAAGAACC

TCTACGAAAGTGGCCTTAATTCACTGAACCCACGAAAGCTAC

>Bathygadus_antrodes

GCGGTTATACGAGAGGCCCAAGTTGATGAAAACGGCGTAAAGAGTGGTTAAGAAAATAAA

TAAAATAAGGCCGAATAGTCTCATTGCAGTTATACGTTTACGAGACCACTAAGCACATTC

ACGAAAGTAGCCTTAGCATAATTCTGACTCCACGAGAGCCA

>Coelorinchus_gilberti

GCGGTTAAACGGGAGACCCAAGTTGATAGACAACGGCGTAAAGCGTGGTTAAGATATTAA

TTAAATAGGGTTAAATGGCCTCTAAGCAGTCAAATACTATCGAAGCCACAAAGCACATCT

ACGAAAGTAACCCTAGTTCTTATGACCCCACGAAAGCCA

>Coelorinchus_japonicus

GCGGTTAAACGGGAGACCCAAGTTGATAAACAACGGCGTAAAGCGTGGTTAAAGTATTAA

TTAAATAGGGTCAAATAGCCTCTAAGCAGTCAAATACTATCGAGACCACAAAGTACACCT

ACGAAAGTGACCCTAATACTCCTGACCCCACGAAAGCCA

>Coelorinchus_kishinouyei

GCGGTTAAACGGGAGACCCAAGTTGATAGACAACGGCGTAAAGCGTGGTTAAAGTATTAA

TTAAATAGGGTCAAATAGCTTCTAAGCAGTCAAATACTATCGAAGCCACAAAGCACACTC

ACGAAAGTAACCCTAGTACCCCTGACCCCACGAAAGCCA

>Coelorinchus_macrochir

GCGGTTAAACGGGAGACCCAAGTTGATAGACAACGGCGTAAAGCGTGGTTAAAGTACTAA

TTAAAATAGGGTCAAATAGCCTCTAAGCAGTCAAATACTATCGAGACCACAAAGCACATC

TACGAAAGTAACCCTAATACCCCTGACCCCACGAAAGCCA

>Macrouridae_sp.

GCGGTTAAACGGGAGGCCCAAGTTGAAAGACAGCGGCGTAAAGCGTGGTTAAGGTGCTAT

ATTAAATAGGGCCGAATAGTCTCAAAGCAGTCAAATCTATTCGAGACCACGAAGCACATC

CACGAAGGTGGCCCTAACACCCCTGACCCCACGAAAGCCA

>Coryphaenoides_marginatus

GCGGTTAAACGGGAGACCCAAGTTGACAGACGACGGCGTAAAGCGTGGTTAAGATACTTA

TTAAATAGGGCCGAATAGTCTCAAAACAGTCAAATCTATTCGAGACCACAAAGTTCATCC

ACGAAGGTGGCCCTAAACACCTCTGACCCCACGAAAGCCA

>Coryphaenoides_nasutus

GCGGTTAAACGGGAGACCCAAGTTGACAGACGGCGGCGTAAAGCGTGGTTAAGATACTTA

CTAGATAGGGCCGAATAGTCTCAAAACAGTCAAATCTATTCGAGACCACGAAGCACATCC

ACGAAGGTGGCCCTAATACCCCTGACCCCACGAAAGCCA

>Ventrifossa_garmani

GCGGTTAGACGGAGGGCCCAAGTTGATAAACAGCGGCGTAAAGCGTAGTTAAGATAACAA

ACAAATAGGACCGAATACTCTCAAAGCAGTTAAATGCATTCGAGACCACTAAGCACACCC

ACGAAAGTGACCCTAATAGTTCTGACCCTACGAAAGCTA

>Halargyreus_johnsonii

GCGGTTATACGAGAGACCCAAGTTGATGAAAAACGGCGTAAAGCGTGGTTATGTAAACTA

AATAATAGGGCCGAACGGCCTCAAAGCAGTTATACGCACTCGAAGTCACGAAGTACAATT

ACGAAAGTAGCCCTACCAACCTCTGATCCCACGAAAGCCA

>Laemonema_longipes

CGGTTATACGAGAGACCCAAGTTGATAAAAAACGGCGTAAAGCGTGGTTAAGAAAATTAA

ACTATAGGGTTGAATGGCCTCAATTTAGTTAAATGCACTCGAAGCCGTGAAGCACAATTA

CGAAAGTAGCCCTACCAAATCTCTGACTCCACGAAAGCCA

>Lepidion_inosimae

GCGGTTATACGAGAGGCCCAAGTTGATGAAAAACGGCGTAAAGCGTGGTTAAGAAAATAA

GATAATAGGGCCGAATGGCCTCAAAGCAGTTATACGCACTCGAAGTCTCGAAGCACAATC

ACGAAAGTAGCCCTAACCTGCCTCTGATTCCACGAAAGCCA

>Hoplobrotula_armata

GCGGTTATACGAGTAGGCCCAAGTTGATATAAAACGGCGTAAAGCGTGGTTAGGGGGCTC

CTATAAACTAAAGCCGAACACCCCCAGAGCTGTTATACGCATCCGGGGGCATGAAGAACC

ACCACGAAAGTGACTTTACCCCGCCCGAACCCACGAGAGCTAA

>U98.5_Ophidiidae_sp.

CGGTTATACGAGTAGGCCCAAGTTGATAAACAGCGGCGTAAAGCGTGGTTAGGGAAGTAA

CCAAACTAAAGCCGAACACCCTCAGAGCTGTTATACGCATCCGAGGGCATGAAGTACCAC

CACGAAAGTGGCTTTACCCCACCCCGAACCCACGAAAGCTA

>Hypoatherina_valenciennei

GCGGTTATACGAGAGGCCCAAGTTGATAGCCATCGGCGTAAAGAGTGGTTAAGAAAACCC

CTAAAACTAAAGCTGAACACTCTCAAAACTGTTATACGTACCCGAGAGCAAGAAGCCCTT

CTACGAAAGTGGCTTTAACCCTTCTGAACCCACGAAAGCTG

>Strongylura_anastomella

CGGTTAGACGAGAGGCCTAAGTTGATAAACACCGGCGTAAAGAGTGGTTAGGAAATAACC

TAGAACTAAAGCCAAATATTCTCATAGCTGTTATACGCACCCGAGAATAAGAAGCCCCCT

TACGAAGGTAGCTTTAATTTATCCGACTCCACGAAAGCTG

>Melamphaidae_sp.

CGCGGTTATACGAGCGACCCAAGCTGATAGTCATCGGCGTAAAGAGTGGTTAAGGGATAA

CATAAACTAAAGCCGAACGCCCTCAAGGCTGTTATACGCACCCGAGAGTACGAAGCCCCA

TTACGAAAATGACTTTAAACCCCACCTGATCCCACGAAAGCTA

>U98.5_Scopeloberyx_robustus_gotu1

CGGTTATACGAGCGACCCAAGCTGATAGCCCCCGGCGTAAAGAGTGGTTAAGGATAAATT

AAACTAAAGCCGAACGCCCTCAAAGCTGTTATACGCACTCGAGGGTACGAAGCCCCATCA

CGAAAGTGGCTTTAACCCCCACCTGACCCCACGAAAGCTA

>Gephyroberyx_sp.

CGCGGTTATACGAGAGGCCCAAGTTGATAATCAACGGCGTAAAGAGTGGTTAAGGAAACA

CCCGCAACTAAAGCCGAACACCCTCAGAGCTGTTATACGCACCCGAGGGCATGAAGTACC

ACCACGAAAGTGGCTTTACTTTTCCTGAACCCACGAAAGCTA

>Hoplostethus_cf._crassispinus

CGGTTATACGAGAGACCCAAGTTGATAAGCAACGGCGTAAAGAGTGGTTAAGGAAACACC

CCAACTAAAGCCGAACATTCTCAGAGCTGTTATACGCACCCGAGGACATGAAGCACCACT

ACGAAAGTGGCTTTACCTTTCCTGAACCCACGAAAGCTATGG

>Hoplostethus_japonicus

GCGGTTATACGAGAGACCCAAGTTGATAAGCAACGGCGTAAAGAGTGGTTAAGGAAACAC

CCAAACTAAAGCCGAACATTCTCAGAGCTGTTATACGCACCCGAGAACATGAAGCACCAC

TACGAAAGTGGCTTTACCTTTCCTGAACCCACGAAAGCTA

>Beryx_splendens

CGGTTATACGAGCGACCCAAGCTGATAGTCACCGGCGTAAAGAGTGGTTAGAGGACAGCC

CAAACTAAAGCCGAACGCCCTCAAAGCTGTTATACGCATCCGAAGGTACGAAGAACCATC

ACGAAAGTGGCTTTACCCCCCACCCGAACCCACGAAAGCTA

>Hozukius_emblemarius

CGGCTATACGAGAGACCCAAGTTGATACCATTCGGCGTAAAGAGTGGTTATGGAAAATAA

AAACTAAAGCCGCACACCTTCAAAGCTGTTATACGCATCCGAAGGCTAGAAGACCAACCA

CGAAGGTAGCTTTACAACCCCTGACCCCACGAAAGCTC

>Sebastes_sp.

GCGGCTATACGAGAGACCCAAGTTGATACCATTCGGCGTAAAGAGTGGTTATGGAAAATA

AAGACTAAAGCCGCACACCTTCAAAGCTGTTATACGCATCCGAAGGCTAGAAGATCAACC

ACGAAGGTAGCTTTACAACCCCTGACCCCACGAAAGCTC

>Lepidotrigla_sp.

CGGTTATACGAGAGGCCCAAGTTGACAGTCACCGGCGTAAAGAGTGGTTAAAGAATAATT

AAAACTAAAGCCGAACACCTTCAAGGCAGTTATACGCACCCGAAGGCCAGAAGCCCAACT

ACGAAAGTGGCTTTATCTTTCCTGAACCCACGAGAGCTA

>Erilepis_zonifer

GCGGTTATACGAGAGGCCCAAGTTGAAAGACATCGGCGTAAAGAGTGGTTAAGTAAAAAT

TAAACTAAAGTCGAACATCTTCAAGGCTGTTATACGCATCCGAAGACAAGAAGTTCAACC

ACGAAGGTGACTTTATTTATACTGAACCCACGAAAGCTAAGACA

>Ebinania_vermiculata

CGGTTATACGAGAGGCCCAAGTTGACAAATACCGGCGTAAAGCGTGGTTAAGTTAAAACT

CCTACTAAAGCCGAACATCTTCAAGGCTGTTATACGCAACCGAAGACAGGAAGTTCAACT

ACGAAAGTGGCTTTATCTGATCTGACCCCACGAAAGCTAA

>Psychrolutes_macrocephalus

GCGGTTATACGAGAGGCCCAAGTTGACAAACACCGGCGTAAGGCGTGGTTAAGTTAAAAC

TCCTACTAAAGCCGAACATCTTCAAGGCTGTTATACGCAACCGAGGACAGGAAGTTCAAC

CACGAAAGTAGCTTTATTTGATCTGAACCCACGAAAGCTA

>Psychrolutes_phrictus

GCGGTTATACGAGAGGCCCAAGTTGACAAACACCGGCGTAAGGCGTGGTTAAGTTAAAAC

TCCTACTAAAGCCGAACATCTTCAAGGCTGTTATACGCAACCGAAGACAGGAAGTTCAAC

CACGAAAGTGGCTTTATCTGATCTGAGCCCACGAAAGCTA

>Allocareproctus_jordani

CGGTTATACGAGAGGCCCAAGTTGAAAGACACCGGCGTAAAGCGTGGTTAAGTTAAAACT

AAACTAAAGTCAAATATCTTTAGGGCTGTTACATGTATCCGAAGACGGGAGCCTCAATCA

CGAAAGTAACTTTATGTAAACTGAACCCACGAAAGCTA

>Paraliparis_cephalus

GCGGTTATACGAGAGGCCCAAGTTGAAAGACACCGGCGTAAAACGTGGTTAAGTTAAAAT

TTAAACTAAAGTTAAACATCTTCAAAACTGTTATACGTATCCGAAGACGGGAGCCTCAAT

CACGAAAGTAACTTTATACAAACTGAACCCACGAAAGCTA

>Paraliparis_meridionalis

GCGGTTATACGAGAGGCCCAAGTTGAAAGACACCGGCGTAAAACGTGGTTAAGTTAAAAT

TTAAACTAAAGTTAAACATCTTCAAAACTGTTATACGTATCCGAAGACAAGAACCTCAAT

CACGAAAGTAACTTTATATAAGCTGAACCCACGAAAGCTA

>U98.5_Elassodiscus_caudatus_gotu1

GCGGTTATACGAAAGGCTCAAGTTGAAAGACACCGGCGTAAAGCGTGGTTAAATTAAAAT

TTAAACTAAAGTTAAACATCTTCAAGACTGTTATACGTACCCGAAAATGAGAACCTCAAC

CACGAAAGTAACTTTATGTAAACTGAATCCACGAAAGCTA

>U98.5_Percichthyidae_sp.

GCGGTTATTCGAGAGGCTCAAGTTGAGAGAACCACGGCGTAAAGAGTGGTTAAGATAAAT

TAAAAAATAAAGCCAAACCCCCTCCACACTGTGATACGCCCCCGAGGGGAGGAAGCCCAA

TCACGAAAGTGGCTTTACAACTCCTGAACCCACGAAAGCTA

>Malakichthys_sp.

GCGGTTATACGAGGGGCCCAAGTTGATAGCCACCGGCGTAAAGAGTGGTTAGGATAAATA

AAATACTAAAGCCGAACGCCCTCAGAGCTGTTATACGCCCCCGAAGGTAAGAAGATCAAT

CACGAAAGTGGCTTTATAATGACTGAACCCACGAAAGCTA

>Acropomatidae_sp.

CGGTTATACGAGAGACCCAAGTTGATGCACACCGGCGTAAAGAGTGGTTAGTCAAGACTT

TTAAAATAAAGCCGAACACCCTCAGAGCTGTTATAAGCATCCGAAGGTACGAAGTTCAAT

CACGAAAGTAGCTTTAAATCACATGACCCCACGAAAGCTA

>Epigonus_denticulatus

GCGGTTATACGAGAGACCCAAGTTGACAGACATCGGCGTAAAGCGTGGTTAAGACAAATT

GAAGACTAAAGCCGAACCCCCTCAAAGCTGTTATACGCATCCGAGGGCAAGAAGCTCAAT

CACGAAAGTGGCTTTACCTCCCTGAATCCACGAAAGCTATG

>Alectis_ciliaris

CGGTTATACGAGGGGCTCGAGTTGACAGACAACGGCGTAAAGAGTGGTTAAGGAAAATAT

TCAACTAAAGCGGAACGCCCTCACAGCTGTTATACGTTTCCGAGGGTATGAACCCCAACT

ACGAAAGTGGCTTTACACTACCTGAACCCACGAAAGCTA

>Decapterus_tabl

CGCGGTTATACGAGAGGCTCAAGTTGACAGACAACGGCGTAAAGAGTGGTTAAGGAAAAC

ATCCAACTAAAGCGGAACCCTCTCACTGCTGTTATACGCTTCCGAGAGGATGAACCACAA

CTACGAAGGTGGCTTTATAAAACCCGAACCCACGAAAGCTA

>Pagrus_major

CGGTTATACGGGAGGCCCAAGTTGTTAAAAATCGGCGTAAAGGGTGGTTAAGAGCAAGCT

TAAAATTAAAGCCGAACGCCTTCTAGGCTGTTATACGCATCCGAAGGTAAGAAGCCCAAT

CACGAAAGTAGCTTTATATTTTCTGACCCCACGAAAGCTA

>Rhabdosargus_sp.

CGGTTATACGGGTGGCCCAAGTTGTCAGAAGTCGGCGTAAAGGGTGGTTAAGAGCAAACT

TAAGATTAAAGCCGAACACCTTCCAGACCGTTATACGTATCCGAAGGCAAGAAGCTCAAT

TACGAAAGTAGCTTTATATTTTCTGAACCCACGAAAGCTA

>Sciaenidae_sp.

GCGGTTATACGAGAGGCCCAAGTCGATAGTCCACGGCGTAAAGAGTGGTTAGAGAACCCC

AATTACTAAAGCCGAACCCCTTCAAGGCTGTTATACGCACACCGAAGAGGAGAAGCCCGC

CCACGAAAGTGGCTTTACAATCTTGAACCCACGAAAGCTA

>Pennahia_argentata

GCGGTTATACGAGAGGCCCAAGTCGATAGTCAACGGCGTAAAGAGTGGTTAGAGAAACCC

CGTTACTAAAGCCGAACCCCTTCAAGGCTGTTATACGCTTACCGAAGAGGAGAAGCCCAC

CTACGAAAGTAGCTTTATAATCTTGAACCCACGAAAGCTA

>U98.5_Zoarcidae_sp.

CGGTTATACGAGAGGCCCAAGTTGAAAGACCCCGGCGTAAAGAGTGGTTAAGCTAGAACT

CATACTAAAGCCGAACATCCCCACGGCTGTTATACGCACCCGAAGACAAGAAGCTCAGCC

ACGAAGGTGGCTTTATTTGCCTGAACCCACGAAAGCTA

>Pseudoscopelus_sagamianus

GCGGTTATACGGGTGGTCCAAGTTGACAGACACCGGCGTAAAGCGTGGTTAAGAAATACT

GAAACTAAAGCCGAACGCCCTCAAAGCAGTTATACGCATCCGAAGGTATGAAGCCCCACC

ACGAAAGTGGCTTTACAAAGTCTGACTCCACGAAAGCTA

>Uranoscopus_japonicus

CGGTTATACGAGAGGCTCAAGCTGATAGCCCAACGGCGTAAAGAGTGGTTAAGGGAGTAT

TTTCAATAAAGCCGAGAATCAACAAAGCAGTTATACGCACCCGAAAAAAAAAGACCGATC

ACGAAAGTAGCTTTAACACCCCTGAGCCCACGAAAGCTAG

>Sphyraena_pinguis

GCGGTTATACGAGAGGCCCAAGTTGACAACCGACGGCGTAAAGCGTGGTTAGGGGAAATA

TAAACTAAAGCCGAACGCCCCCAATGCTGTCTAATGCTTCGAGGGTATGAAGAACATCGA

CGAAAGTGGCTTTATGACACCTGAACCCACGAAAGCTG

>Gempylus_serpens

CGGTTATACGAGAGGCCCAAGTTGACAGAAGCCGGCGTAAAGAGTGGTTAAGGAAAATAC

AAACTAAAGCCGAACGCCTTCAGAGCAGTTATACGCACCCGAAGGTATGAAGCCCCACTA

CGAAAGTGGCTTTACACCCCCTGACCCCACGAAAGCTAT

>Nealotus_tripes

CGGTTATACGAGCGACCCAAGTTGACAAACCCCGGCGTAAAGCGTGGTTAGGGAAACCCA

AAAACTAAAGCCGAATGCCTTCAAGGCAGTTATACGCAATCCGAAAATACGAAGCCCCAC

CACGAAAGTGGCTTTACCACCCCTGACTCCACGAAAGCTA

>Promethichthys_prometheus

GCGGTTATACGAGTGGCCCAAGTTGATAAAAACCGGCGTAAAGCGTGGTTAAGGTCATAC

TACAAACTAAAGCCGAACCTCCTCACAGCAGTTATACGCTTATGAAGAAACTGAAGCTCC

CCCACGAAAGTGGCTTTACTACCCCACCTGACCCCACGAAAGCTA

>Rexea_prometheoides

GCGGTTATACGAGGGGCCCAAGTTGACAGAAAACGGCGTAAAGCGTGGTTAAGGAAAAAC

TTAAACTAAAGCCGAATGACTTCAGAGCAGTTCAAAGCATCCGAAAACACGAAGCCCACC

CACGAAAGTGGCTTTACTACCCCTGACTCCACGAAAGCTAT

>Ruvettus_pretiosus

CGGTTATACGAGAGGCCCAAGTTGACAGACACCGGCGTAAAGCGTGGTTAAGGAAAACTT

AAACTAAAGTCGAACACCTTCAAAGCAGTTATACGCACCCGGAGGTACGAAGCCCCACCA

CGAAAGTGGCTTTATTACCCCTGACCCCACGAAAGCTA

>Trichiuridae_sp.

CGGTGATACGAGAGGCCCGAGCTAACAGCCCCCGGCGTAAAGCGTGGTTAGGGTAAGTCA

AAACTAAAGCCGAACGCCCCTAGGGCCGTGATACGCGTATAAAGGTATGAAGTCCCACCA

CGAAAGTGGCTTTAATACTTCCTGACCCCACGAAAGCTAAGAT

>Auxis_rochei

CGGTTATACGAGAGGCCCAAGTTGACAGACACCGGCGTAAAGCGTGGTTAAGGAAAACTA

AAACTAAAGCCGAACACCTTCAGGGCAGTTATACGCATCCGAAGGCACGAAGCCCCACCA

CGAAAGTGGCTTTATGACCCCCTGACCCCACGAAAGCTA

>Katsuwonus_pelamis

CGGTTATACGAGAGGCCCAAGTTGACAGACACCGGCGTAAAGCGTGGTTAAGGTAAACTA

ACACTAAAGCCGAACACCTTCAGGGCAGTTATACGCATCCGAAGGCACGAAGCCCCACCA

CGAAAGTGGCTTTATGACCCCTGACCCCACGAAAGCTAT

>Scombridae_sp.

GCGGTTATACGATAGGCCCAAGTTGACAGACCCCGGCGTAAAGCGTGGTTAGGGAAAACT

CAAAACTAAAGCCGAATATCTTCAGGGCAGTTATACGCTTCCGAAGACACGAAGCCCTTC

CACGAAAGTGACTTTATTACCCCCGACCCCACGAAAGCTA

>Scomberomorus_niphonius

GCGGTTATACGAGAGGCCCAAGTTGACAACCACCGGCGTAAAGCGTGGTTAAGATATAAT

CAAAACTAAAGCCGAATGTTTTCAAGGCAGTCATACGCTTCCGAAGACACGAAGCCCCAC

CACGAAAGTGGCTTTAACAATCCCTGAACCCACGAAAGCTA

>Thunnus_sp.

CGCGGTTATACGAGAGGCCCAAGTTGACAGACACCGGCGTAAAGCGTGGTTAAGGTACAC

GAAAACTAAAGCCGAACACCTTCAGGGCAGTTATACGCATCCGAAGGCACGAAGCCCCAC

CACGAAAGTGGCTTTATGAACCCTGACCCCACGAAAGCTATG

>Symphurus_cf._microrhynchus

GCGGTTATACGAGAAACTCTAGTTGATAATGCACGGCGTAAAGGGTGGTTAGGAACAATA

AATGAAGTAAGGCCTAACGATTTTTATGCTGTTATAAGTTTACAAAAATTAGAAGCCCGT

CCACGAAGGTGGCCTTATTACCTCTGACCCCACGAAGGCTTG

>Triacanthus_biaculeatus

GCGGTTATACGAGGGACCCAAGTTGATATTCGCCGGCGTAAAGAGTGGTTAAGACATACA

ATGAAACTAAGGCGGAATTTCTTCACAGTCGTCATACGCTTTTGGAGATAAGAAACCCAA

TAACGAAAGTAGCCTTATGATATCCGAATCCACGAAAGCTA

>Arothron_firmamentum

CGGTTATACGAGAGACCCAAGTTGTTAGCCCCCGGCGTAAAGAGTGGTTAAAAACACAAC

ATTAAACTGAGGCCGAACATCCTCAAGGCAGTTATACGCTCCCGAGGATATGAAGAACAA

TTACGAAAGTAGCCTCACCCCCTTTGAACCCACGAAAGCTAGG
